# Implementation of Telemental Health Services Before COVID-19: Rapid Umbrella Review of Systematic Reviews

**DOI:** 10.2196/26492

**Published:** 2021-07-20

**Authors:** Phoebe Barnett, Lucy Goulding, Cecilia Casetta, Harriet Jordan, Luke Sheridan-Rains, Thomas Steare, Julie Williams, Lisa Wood, Fiona Gaughran, Sonia Johnson

**Affiliations:** 1 Centre for Outcomes Research and Effectiveness Division of Psychology and Language Sciences University College London London United Kingdom; 2 NIHR Mental Health Policy Research Unit Division of Psychiatry University College London London United Kingdom; 3 King's Improvement Science Centre for Implementation Science King's College London London United Kingdom; 4 NIHR Applied Research Collaboration King's College London London United Kingdom; 5 Institute of Psychiatry, Psychology and Neuroscience King's College London and South London and Maudsley NHS Trust London United Kingdom; 6 Centre for Implementation Science Health Service and Population Research Department King's College London London United Kingdom; 7 Division of Psychiatry University College London London United Kingdom; 8 Camden and Islington NHS Foundation Trust London United Kingdom

**Keywords:** umbrella review, remote, telemental health, telepsychiatry, video-based therapy, COVID-19, remote therapy, review, mental health, therapy, eHealth, telemedicine, mobile phone

## Abstract

**Background:**

Telemental health care has been rapidly adopted for maintaining services during the COVID-19 pandemic, and a substantial interest is now being devoted in its future role. Service planning and policy making for recovery from the pandemic and beyond should draw on both COVID-19 experiences and the substantial research evidence accumulated before this pandemic.

**Objective:**

We aim to conduct an umbrella review of systematic reviews available on the literature and evidence-based guidance on telemental health, including both qualitative and quantitative literature.

**Methods:**

Three databases were searched between January 2010 and August 2020 for systematic reviews meeting the predefined criteria. The retrieved reviews were independently screened, and those meeting the inclusion criteria were synthesized and assessed for risk of bias. Narrative synthesis was used to report these findings.

**Results:**

In total, 19 systematic reviews met the inclusion criteria. A total of 15 reviews examined clinical effectiveness, 8 reported on the aspects of telemental health implementation, 10 reported on acceptability to service users and clinicians, 2 reported on cost-effectiveness, and 1 reported on guidance. Most reviews were assessed to be of low quality. The findings suggested that video-based communication could be as effective and acceptable as face-to-face formats, at least in the short term. Evidence on the extent of digital exclusion and how it can be overcome and that on some significant contexts, such as children and young people’s services and inpatient settings, was found to be lacking.

**Conclusions:**

This umbrella review suggests that telemental health has the potential to be an effective and acceptable form of service delivery. However, we found limited evidence on the impact of its large-scale implementation across catchment areas. Combining previous evidence and COVID-19 experiences may allow realistic planning for the future implementation of telemental health.

## Introduction

### Background

The use of technologies such as phone or video calls between mental health professionals and other mental health professionals, patients, service users, family members, or carers to deliver mental health care (telemental health) has become an essential tool in recent months; importantly, it has taken a central role internationally in maintaining mental health services during the COVID-19 pandemic [1]. Policy makers and mental health professionals, along with mental health service users, now express interest in continuing some use of these technologies long term, even in the absence of pandemic-related social distancing requirements [1-3]. The potential benefits of remote technologies extend beyond adaptation to government social distancing guidelines, allowing the efficiency and flexibility of mental health services to be maximized. The mobilization of telemental health during the pandemic has happened largely ad hoc, thereby achieving remarkably rapid but highly variable implementation. This emergency response has mostly occurred without systematic references to the literature. To plan the effective and acceptable deployment of telemental health beyond the pandemic, it is crucial that we now review all the relevant evidence regarding potential impacts, challenges, and outcomes of widespread remote technology utilization and identify the key mechanisms for its acceptable integration into routine care [4].

Telemental health offers a number of potential benefits that generate a significant interest among service providers not only during the pandemic but also in the long term. For service users across a range of populations, settings, and conditions [5], potential benefits include convenience and improved accessibility, particularly where issues such as physical mobility difficulties, anxiety, or paranoia impede a face-to-face contact [1]. Potential advantages for staff include reduced environmental impact, greater convenience with opportunities for working at home, and ease of communication within and between mental health teams [2]. Although some have argued that problems with building rapport [6] along with privacy and safety concerns may hinder the implementation of remote care, service users have been found to report such apprehensions less than clinicians [7]. Several studies have also suggested that telemental health may be more cost-effective than face-to-face delivery [7].

Despite potential benefits and a substantial body of relevant research, the delivery of remote working remained very limited in most countries before the pandemic with substantial implementation barriers [8], along with the potential for inequalities to be exacerbated. Digital exclusion is an important concern for service users who lack the necessary skills, space, equipment, and monetary resources to access web-based treatment. This is more marked in marginalized groups such as people from BAME (Black, Asian, and minority ethnic) and low socioeconomic status backgrounds [9]. Further risks include the loss of privacy and deterioration in therapeutic relationships [1,10-12]. Staff participation is also impeded by technological and environmental difficulties, and they express reservations regarding the quality of assessments, deterioration of therapeutic relationships, and limitations in the extent to which there is a focus on physical as well as mental health [8,11,12].

### Objectives

Therefore, the potential benefits and disadvantages of telemental health are finely balanced. Risks of longer-term rollout of telemental health without close attention to intended and unintended consequences include the digital exclusion of some of those already most disadvantaged and decline in the quality of care and, potentially, of outcomes. One source with the potential to inform policy makers and service planners in their future telemental health strategies is the substantial body of research studies published before the pandemic. This paper therefore aims to provide a rapid summary of the existing literature on the effectiveness, cost-effectiveness, barriers and facilitators for implementation, acceptability, and reach of remote interventions for the assessment and treatment of mental health problems. Our objective is to identify, appraise, and synthesize the systematic reviews of literature and guidance on telemental health, including qualitative and quantitative outcomes using the *umbrella review* or *review of reviews* methodology. Umbrella reviews are useful in summarizing a broad evidence base to inform policy [13]. It is hoped that the results will help illuminate the benefits and remaining challenges while implementing telehealth technologies during the remainder of the pandemic and in the perhaps permanently changed reality that follows.

## Methods

### Overview

A rapid umbrella review was conducted guided by the World Health Organization practical guide for Rapid Reviews to Strengthen Health Policy and Systems [14] and adhering to PRISMA (Preferred Reporting Items for Systematic Reviews and Meta-Analyses) guidelines [15] and umbrella review guidance [16]. In line with the agreed rapid review methodology, this review aims to provide a timely but robust answer to the research question by accelerating some aspects of the systematic review process while maintaining transparency and protocol-driven decision making throughout [14]. The protocol was prospectively registered on PROSPERO (CRD42020208085).

### Search Strategy and Selection Criteria

#### Overview

The search strategy implemented a combination of keywords and subject heading searches across PsycINFO (01/01/2010-26/08/2020), PubMed (01/01/2010-26/08/2020), and the Cochrane Database of Systematic Reviews (01/01/2010-26/08/2020). Searches combined terms for systematic reviews, mental health disorders, and telemental health. The full search strategy is available in Multimedia Appendix 1. We included systematic reviews that met the following criteria.

#### Population

Staff working within the field of mental health, people receiving mental health care or with mental health diagnoses, family members, or carers of people receiving mental health care were included. In line with our focus on people whose conditions tend to make them eligible for telemental health care, we also included people with dementia, neurodevelopmental disorders, and addiction but excluded people with primary sleep disorders, unless combined with other mental health problems.

#### Interventions

Any form of spoken or written communication conducted between mental health professionals and patients, service users, family members, carers, or other mental health professionals using either the internet or the telephone were included. We excluded the reviews of digital interventions where the primary aim of the technology was not to facilitate direct therapeutic contact with a mental health professional; thus, for example, we excluded apps and websites delivering assessment or treatment in a digital format.

#### Outcomes

Reviews reporting at least one of the following: implementation outcomes (outcomes relating to the process of care, adherence to intended models, uptake and coverage, and barriers and facilitators to implementation), acceptability outcomes (including staff and service user satisfaction, and experiences of the therapeutic relationship and communication), clinical effectiveness, cost-effectiveness, or evidence-based guidance for telemental health were included. Qualitative and quantitative data were analyzed.

#### Design

Systematic reviews with or without meta-analyses, realist reviews, and qualitative meta-syntheses were included. We considered reviews to be of sufficient quality for inclusion if they searched at least three databases based on recommendations by Cochrane and the Assessment of Multiple Systematic Reviews (AMSTAR) [17], meaning that at least two databases plus subject-specific databases were searched. In line with recommendations for conducting systematic reviews for quantitative data [15], quantitative reviews were also required to include appraisal of the quality of included studies.

Owing to the rapid nature of the review, we limited our search to reviews published since January 2010 and those available in the English language. This was a pragmatic decision because studies published before 2010 would still be picked up within systematic reviews.

A total of 3 reviewers (PB, LG, and CC) double screened 10% of titles and abstracts, and disagreements were discussed until consensus was reached. The remaining titles were independently screened, and studies not meeting the inclusion criteria were excluded. Full-text articles were subsequently reviewed by 5 reviewers (PB, TS, LG, CC, and LW). A selection of full texts was double checked to ensure consistency, and any reviews that did not facilitate a straightforward inclusion or exclusion decision were discussed with the wider review group.

### Data Extraction

A total of 7 reviewers (LG, CC, PB, TS, LSR, JW, and HIJ) extracted data from the included reviews using a Microsoft Excel-based form. In total, 10% of extractions were double checked by a second reviewer, and inconsistencies were discussed and corrected. The extracted data included citation details, objectives, type of review, participant details (including gender, ethnicity, age, and mental health diagnosis accompanied by staff details where relevant), types of telemental health intervention reviewed, setting and context (mental health service, community, inpatient or residential, or primary mental health care service), number of databases sourced and searched, date range of database searching, the publication date range of studies included in the review informing each outcome of interest, number of included studies, types of studies and country of origin of studies included, instrument used to appraise the primary studies and the rating of their quality; reported clinical, cost-effectiveness, and implementation outcomes; method of synthesis or analysis employed to synthesize the evidence; and conclusions of the review authors.

### Quality Assessment

The quality of each included systematic review was assessed using the AMSTAR 2 checklist [17]. This is a revised version of the original AMSTAR checklist [18], which is a validated measure of systematic review quality [19]. The checklist was used to give each review an overall rating of quality ranging from high (0 or 1 noncritical weakness: providing an accurate and comprehensive summary of the results of the available studies that address the question of interest) to critically low (more than 1 critical flaw with or without noncritical weaknesses: the review should not be relied upon to provide an accurate and comprehensive summary of the available studies) [17]. The study quality was assessed along with data extraction. Table 1 presents the quality ratings.

**Table 1:** Study characteristics.

Study	Intervention type (number of studies)	Comparator (number of studies)	Search dates	Number of studies included	Study designs (number of studies)	Patients included, n (% female)	Diagnoses (number of studies)	Population age in years, mean (range)	Ethnicity, n (%)	Countries covered (number of studies)	Quality appraisal rating (AMSTAR 2^a^)
Harerimana et al (2019) [20]	Mobile apps (NR^b^); smart technologies (NR); teleconferencing systems (NR); internet-based therapies (NR); Skype (videoconferencing) calls (NR)	Waiting list or TAU^c^ (NR); no comparator (NR)	1946-2017	9	Pilot RCT^d^ (n=2); RCT (n=2); program case analysis (n=1); quasiexperimental study (n=1); prospective design (n=1); cross-sectional survey (n=1); case study (n=1)	2032 (NR)	Depression or self-reported depressive symptoms (n=9)	>65 (NR)	NR	United States (n=5); Australia (n=1); Canada (n=1); China (n=1); The Netherlands (n=1)	Low
Dorstyn et al (2013) [21]	Telecounseling, that is, telephone, videophone, and computer (NR) or web-based digital media, that is, email, audio-only, or audio-video communication via the internet (NR)	TAU (n=3); face-to-face (n=1); minimal support or waitlist (n=2); no comparator (n=2)	1970-2013	9 (8 different samples)	RCT (n=7); single arm (n=1); nonrandomized controlled trial (n=1)	498 (n=66)	Depression or psychiatric comorbidities with depression symptoms (n=9)	54 (NR)	Hispanic, 243 (52); Latino, 139 (30); Asian, 105 (21); African American, 11 (2)	United States (n=6); Canada (n=1); Australia (n=1)	Critically low
Berryhill et al (2019a) [22]	Video-based CBT^e^ (n=12); video-based behavioral activation (n=5); video-based acceptance and behavioral therapy (n=1); video-based exposure (n=3); video-based metacognitive therapy (n=1); video-based problem-solving therapy (n=2); video-based therapy in multiple modalities (n=9)	Face-to-face psychotherapy (n=16); face-to-face or telephone (n=2); no control (n=15)	1991-2017	33	RCT (n=14); quasiexperimental (n=4); single cohort study—before and after (n=9); case study (n=4); multiple baseline design (n=1); single case interrupted time series (n=1)	NR	Depression (n=9); PTSD^f^ (n=12); depression with comorbid anxiety or PTSD (n=12)	NR (10.3-80.4)	NR	NR	Critically low
Berryhill et al (2019b) [23]	Video-based CBT (n=12); video-based behavioral activation (n=3); video-based ACT^g^ (n=1); video-based exposure therapy (n=2); video-based problem-solving therapy (n=1); video-based metacognitive therapy (n=1); multiple modality (n=1)	Face-to-face psychotherapy (n=20); no control (n=1)	1991-2017	21	RCT (n=6); quasiexperimental (n=4); uncontrolled (n=11)	NR	Depression (n=2); PTSD (n=7); anxiety disorder (ie, PD^h^, GAD^i^, and social phobia; n=5); depression or mood disorder (n=7)	NR (8-62)	NR	United States (n=10); Australia (n=6); Canada (n=5)	Critically low
Bolton and Dorstyn (2015) [24]	Internet-based CBT with therapist support via telephone calls, introductory face-to-face meetings, or emails (n=6); video-based CBT (n=5)	Face-to-face (n=5); supportive counseling (n=1); waitlist (n=1); no comparator (n=4)	1970-2014	11	RCT (n=4); nonrandomized (n=7)	472 (NR)	PTSD (n=11)	40 (18-68)	NR	United States (n=6); Australia (n=3); Canada (n=1); United Kingdom (n=1)	Critically low
Christensen et al (2019) [25]	Video consultations and telepsychiatry (n=21)	Face-to-face (n=11); no control (n=10)	2000-2017	21	RCT (n=7); surveys (n=3); intervention study (n=6); evaluation using qualitative and quantitative methods (n=1); qualitative studies (n=4)	2525 (NR)	Depression (n=6); various diagnoses (n=15)	NR	NR	United States (n=12); Canada (n=5); Spain (n=1); Australia (n=1); Hong Kong (n=1); Germany (n=1)	Low
Coughtrey and Pistrang (2018) [26]	CBT (n=12); ERPT^j^ (n=1); behavioral therapy (n=1)	Face-to-face exposure response therapy (n=1); telephone emotion-focused therapy (n=1); TAU (n=5); waitlist (n=3); no comparator (n=4)	1991-2016	14	RCT (n=9); uncontrolled design (n=3); quasiexperimental (n=2)	750 (NR)	Depression (n=10; 5 with physical comorbidities); OCD^k^ (n=2); anxiety disorders (n=2)	NR (32-66)	NR	United States (n=11); United Kingdom (n=2); Canada (n=1)	Low
Drag et al (2016) [27]	Videoconference (n=24)	Face-to-face (n=23); no comparator (n=1)	2000-2015	26	RCT (n=26)	Analysis of assessment, 765 (NR); analysis of efficacy 2097 (NR)	Analysis of assessment: multiple diagnoses (n=6); Alzheimer disease (n=2); schizophrenia (n=3); autism (n=1); analysis of efficacy: multiple diagnoses (n=2); PTSD (n=3); ADHD^l^ (n=1); major depression (n=6); Alzheimer disease (n=1); eating disorders (n=1)	Analysis of assessment, NR (9-68); analysis of efficacy, NR (9-65)	NR	United States (n=17); Canada (n=2); Japan (n=2); China (n=1); New Zealand (n=1); India (n=1); Norway (n=1); Spain (n=1)	Low
Garcia-Lizana and Munoiz-Mayorga (2010) [28]	Videoconference (n=10)	NR	1997-2008	11	RCT (n=10)	1054 (NR)	Multiple diseases (n=4); depression (n=2); panic disorder (n=1); PTSD (n=1); bulimia (n=1); schizophrenia (n=1)	NR	NR	United States (n=6); Canada (n=4); Spain (n=1)	Critically low
Hassan and Sharif (2019) [29]	Not specified videoconferencing treatment intervention (n=2); video-based CBT (n=7); video-based psychoeducation (n=2); video-based relapse prevention (n=1); video-based treatment management (n=1); video-based evaluation of competency to stand trial (n=1)	Face-to-face (n=14)	2000-2017	14	RCT (n=14)	1714 (NR)	Multiple (n=4); depression (n=5); panic disorder (n=1); PTSD (n=1); schizophrenia (n=1); bulimia nervosa (n=1); mental incompetency (n=1)	NR	NR	Canada (n=5); United States (n=8); Spain (n=1)	Critically low
Lin et al (2019) [30]	Psychotherapy (n=10); medication (n=3)	Face-to-face psychotherapy (n=7); telephone (n=2); TAU (n=1); no comparator (n=3)	1998-2018	13	RCT (n=7); quasiexperimental (n=1); nonrandomized pilot studies (n=2); retrospective studies (n=3)	5546 (NR–substantial variability in gender reported)	Substance use disorders, including alcohol (n=5), nicotine (n=3), opioid (n=5)	NR (30.5-52; 1 study did not report)	NR	United States (n=10); Canada (n=2); Denmark (n=1)	Moderate
Lins et al (2014) [31]	Telephone counseling (n=9)	Friendly calls (n=3); TAU (n=6)	2000-2008	12	RCT (efficacy: n=9); qualitative study (experience of intervention: n=3)	NR	Depressive symptoms (n=8); anxiety symptoms (n=1)	NR (60-66)	NR	United States (n=8); Germany (n=1); Canada or United States (n=3)	Moderate
Muskens et al (2014) [32]	Telephone diagnostic interviewing (n=16)	Traditional face-to-face diagnostic interviewing	NR (search was conducted in 2012)	16	NR	1001 (NR)	Studies conducted diagnostic interviewing for a range of diagnoses including depression, anxiety, substance misuse, psychotic disorders, autism, PTSD, manic episodes or mania, panic disorder, social phobia, simple phobia, dysthymia. Included studies interviewed for between 1 and 21 disorders	NR (8.92-76.9)	NR	United States (n=10); United Kingdom (n=2); Brazil (n=1); Australia (n=1); Canada (n=1); Iran (n=1)	Moderate
Naslund et al (2020) [33]	Videoconference for psychiatric or neurological assessment or treatment (n=23); videotaping psychiatric histories (n=1); sending clinical information electronically to psychiatrist for diagnosis and treatment plan (n=1); therapy via text messages (n=1)	Face-to-face (n=26)	2000-2018	26	RCT (n=11); observational study (n=10); pre-post study (n=3); quasiexperimental (n=2)	17,967 (NR)	Depression (n=7); general mental disorders (n=7); child mental health (n=4); geriatric mental health (n=4); PTSD (n=2); suicidal ideation (n=1); epilepsy (n=1)	NR	NR	Canada (n=4); Colombia (n=1); United States (n=15); Spain (n=1); Germany (n=1); Australia (n=2); Israel (n=1); Hong Kong (n=1)	Critically low
Norwood et al (2018) [34]	Video-based CBT (n=10)	Face-to-face CBT (n=10)	NR (search took place in 2018)	10	RCT (n=4); non-RCT (n=2); case studies or series (n=3); uncontrolled trial (n=1)	343 (NR)	Depression, anxiety, or mood disorder (n=3); bulimia nervosa or EDNOS^m^ (n=1); PTSD (n=2); OCD (n=1); panic disorder with agoraphobia (n=1); social anxiety (n=1); NR (n=1)	NR	NR	United States (n=6); Canada (n=1); France (n=1); United Kingdom (n=1); Australia (n=1)	Moderate
Olthuis et al (2016a) [35]	Internet CBT with therapist email or telephone support (n=37); internet behavioral therapy with exposure (n=1)	Waitlist or attentional control (n=20); face to face (n=7); other internet therapies (n=6; multiple control groups (n=5)	Up to 2015	30	RCT	218 (67.1)	Social phobia (n=11); PD with or without agoraphobia (n=8); GAD (n=5); PTSD (n=2); OCD (n=2); specific phobia (n=2); mixed anxiety (n=8)	37.3 (NR)	NR	Sweden (n=18); Australia (n=14); Switzerland (n=3); Netherlands (n=2); United States (n=1)	Moderate
Olthuis et al (2016b) [36]	Internet CBT (with therapist contact) or CBT by phone (n=19)	Face-to-face (n=8); internet-based supportive counseling (n=1); TAU (n=2); telephone (n=1); self-help iCBT^n^ (n=1); waiting list (n=6)	Up to 2016	19	RCT	1491 (67.7)	PTSD (n=13); subclinical PTSD (n=6)	NR	NR	United States (n=13); Sweden (n=3); Germany (n=1); Australia (n=2)	Moderate
Sansom-Daly et al (2016) [37]	N/A^o^ (systematic review of guidelines)	N/A	2004-2014	20	N/A	N/A	N/A	N/A	N/A	United States (n=10); Canada (n=5); Australia (n=1); United Kingdom (n=1); Europe (n=1); South Africa (n=1); New Zealand (n=1)	Low
Turgoose et al (2018) [38]	Video-based exposure (n=10); video-based cognitive processing therapy (n=6); video-based CBT (n=5); mixed interventions (n=11); telephone mindfulness (n=1); video-based behavioral activation (n=2); video-based eye movement desensitization and reprocessing (n=1); video-based anger management (n=2); video-based general coping and psychoeducation interventions (n=3)	Face-to-face (n=41)	Up to 2018	41	NR. A mix of experimental and nonexperimental designs	4130 (NR)	PTSD (n=41)	NR	NR	United States (n=40); Canada (n=1)	Critically low

^a^AMSTAR 2: Assessment of Multiple Systematic Reviews.

^b^NR: not reported.

^c^TAU: treatment as usual.

^d^RCT: randomized controlled trial.

^e^CBT: cognitive behavioral therapy.

^f^PTSD: posttraumatic stress disorder.

^g^ACT: acceptance and commitment therapy.

^h^PD: Parkinson disease.

^i^GAD: generalized anxiety disorder.

^j^ERPT: exposure response prevention therapy.

^k^OCD: obsessive-compulsive disorder.

^l^ADHD: attention-deficit/hyperactivity disorder.

^m^EDNOS: eating disorders not otherwise specified.

^n^iCBT: internet-based cognitive behavioral therapy.

^o^N/A: not applicable.

### Data Synthesis

Heterogeneity in study populations and interventions included in the review, as well as broad inclusion criteria for review study design (eg, qualitative and quantitative), prevented the quantitative pooling of syntheses. As a result, we conducted a narrative synthesis of all interventions and outcomes [39]. This allowed for a more in-depth consideration of all outcome measures and variations in the remote intervention delivery. We grouped reviews by the included population (mental health diagnosis) and further considered the variation in interventions on offer within these subgroups. This was performed for each outcome of interest. Most reviews provided a synthesis of multiple intervention types or failed to adequately differentiate them, making a more thorough comparison across formats impossible.

## Results

### Overview

**Figure 1:**
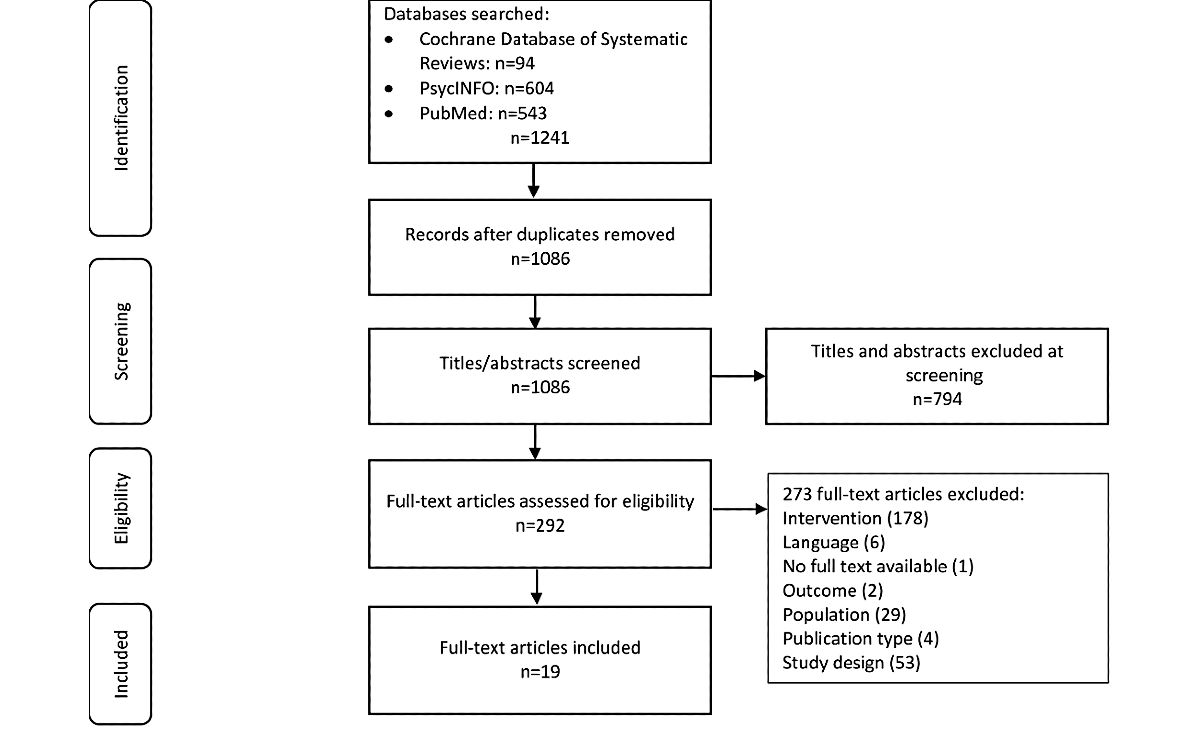
PRISMA (Preferred Reporting Items for Systematic Reviews and Meta-Analyses) diagram.

The search returned 1086 reviews, of which 292 potentially relevant full-text articles were identified. Following full-text checks, 19 reviews met the inclusion criteria (Figure 1), reporting 239 individual studies and 20 guidance documents. In total, 15 of the included reviews examined the clinical effectiveness of telemental health as compared with (1) face-to-face interventions and assessments (n=4), (2) treatment as usual (n=2), or (3) a variety of comparators, including face-to-face, telephone, and treatment as usual (n=9). Eight reviews reported on implementation (broadly defined), including process variables, fidelity, and uptake of interventions, and 10 reviews reported outcomes related to acceptability, including the satisfaction of both service users and clinicians. In total, 1 review focused specifically on the differences in therapeutic alliances between treatment modalities. A total of 2 reviews reported cost-effectiveness, one on this topic only and the other in combination with clinical effectiveness. One review synthesized international guidance on the conduct of videoconferencing-based mental health treatments. Table 1 presents full details of the included reviews, and Figure 1 shows the information on the search and screening process.

Some primary studies were included in more than 1 review: 26 studies appeared in 2 reviews and 27 studies appeared in 3 or more. The remaining 186 studies were included in only 1 review. The double counting of primary studies due to inclusion in multiple reviews contributing to the same outcome was only found for clinical effectiveness outcomes. However, conclusions were similar across reviews, and no review had all the same studies contributing to any synthesis. Multimedia Appendix 2 [20-36,38,40-276] and Multimedia Appendix 3 [40-276] present further details of the study overlap.

### Quality of Included Reviews

Most reviews elicited low confidence in quality appraisal because of multiple weaknesses in the study design. The most common weaknesses were a lack of explicit statements that a protocol was developed before the commencement of the review (explicit statements were reported in two reviews [20,35]), lack of duplicate study selection (duplicate selection was reported in five reviews [28,31,32,35,37]), no report of excluded studies and reasons for exclusion (exclusions were reported in two reviews [31,35]), and no report of sources of funding (sources of funding were reported in three reviews [26,27,31]). Meta-analysis was not performed in the majority of reviews, usually because of heterogeneous data or aims centering around more narrative conclusions such as acceptability (n=12) but in those that included meta-analysis [24,27,31,34-36], all except two [31,34] reviews assessed publication bias. The potential impact of risk of bias was only assessed in two reviews performing meta-analysis [31,34], but all reviews performing meta-analysis used appropriate statistical methods for combining results. The reviews eliciting higher confidence (moderate) were two Cochrane reviews [31,36]. Table 1 presents the quality ratings of reviews, and Multimedia Appendix 4 [20-32,34-38] shows the full details of quality assessments.

### Clinical Outcomes

Clinical outcomes were reported in 15 reviews [20-24,26-31,34-36,38]. Across all patient populations, including patients with anxiety (n=3), posttraumatic stress disorder (PTSD; n=2), depression (n=4) (including in ethnic minorities, n=1 [21], and older adults, n=1 [20]), substance use disorders (n=1), and multiple disorders (n=4), videoconferencing interventions have been reported to cause significant reductions in symptom severity, with outcomes comparable with face-to-face controls where these were included. Telephone-based interventions tended to report similar significant reductions in symptom severity. However, a review of telephone interventions with older adults with depression [20] reported more mixed findings: reductions were reported in emergency room and hospital visits in one study, and in depression in another; however, a third study suggested that telephone interventions did not add to benefit gained from a web-only intervention. Follow-up treatment gains were less widely reported, and conclusions were mixed across the reviews. Although the maintenance of improvements was found at varying follow-up assessments in one review [34] and at 3-6 months in two additional reviews [26,38], two other reviews reported that evidence was inconsistent with some studies reporting that videoconference interventions may show less longevity in the maintenance of effects up to 6 months compared with face-to-face interventions [21,24]. A final review of mixed modality remote interventions suggested that although inferior to face-to-face formats at shorter follow-up, remote interventions may be more beneficial than face-to-face interventions at longer follow-ups (36 months) [20]. Table 2 presents further details on the clinical outcomes.

**Table 2:** Clinical effectiveness outcomes.

Main diagnosis	Study	Intervention (number of studies)	Comparator (number of studies)	Results	Data
**Anxiety**					
	Berryhill et al (2019b) [23]	Video-based CBT^a^ (n=12); video-based behavioral activation (n=3); video-based ACT^b^ (n=1); video-based exposure therapy (n=2); video-based problem-solving therapy (n=1); video-based metacognitive therapy (n=1); multiple modality (n=1)	Face-to-face psychotherapy (n=20); no control (n=1)	In total, 66% (14/21) of studies found statistically significant improvement on validated anxiety measures when videoconferencing psychological therapy was involved. A total of 52% (11/21) of studies reported clinically significant improvements among participants. Controlled study designs comparing face-to-face and videoconferencing psychological therapy sessions (7/10, 70%) found no statistical difference between them.	No combined data were available
	Coughtrey and Pistrang (2018) [26]	Telephone-based CBT (n=2); telephone-based exposure response prevention therapy (n=1); telephone-based behavioral therapy (n=1)	Face-to-face exposure response therapy (n=1); waitlist (n=3)	All 3 RCTs^c^ on anxiety reported significant reductions in anxiety symptoms following telephone-delivered intervention (OCD^d^: comparable reductions with face-to-face treatment, maintained over 6-month follow-up. Panic disorder: significant reductions in panic and gains maintained over 3-month follow-up. Transdiagnostic intervention: significant reductions in anxiety sensitivity, panic, social phobia and PTSD^e^). One quasiexperimental study found significant reductions in OCD symptoms as compared with controls maintained at 12-week follow-up.	RCTs: Cohen *d* range 0.34-1.07 (median 0.69; n=2); uncontrolled: Cohen *d*=1.07 (n=1)
	Olthuis et al (2016a) [35]	Internet CBT with therapist email or telephone support (n=37); internet behavioral therapy with exposure (n=1)	Waitlist or attentional control (n=20); face to face (n=7); other internet-based therapy (n=6); multiple control groups (n=5)	Versus control: therapist-supported iCBT^f^ showed significantly larger improvements in anxiety (n=12), disorder-specific anxiety symptom severity (n=30), and general anxiety symptom severity (n=19) at posttreatment as compared with waiting list, attentional control, information only or internet-based discussion group only controls. Versus unguided iCBT: therapist-supported iCBT showed no difference in improvements in anxiety at posttreatment (n=1), disorder-specific anxiety symptom severity at posttreatment (n=5), and general anxiety symptom severity (n=2) at posttreatment compared with unguided self-help iCBT. Versus face-to-face: therapist-supported iCBT showed no difference in improvements in anxiety at posttreatment (n=4) and 6- to 12-month follow-up (n=3), disorder-specific anxiety symptom severity at posttreatment (n=7) and 6- to 12-month follow-up (n=6) and general anxiety symptom severity (n=6) at posttreatment and at 6- to 12-month follow-up (n=5) as compared with face-to-face CBT.	Waitlist, attentional control, information only or internet-based discussion group–only controls at posttreatment: SMD^g^=–1.06 (95% CI –1.29 to –0.82), *P*<.001; face-to-face CBT at posttreatment: SMD=0.06 (95% CI –0.25 to 0.37); *P*=.36 (no difference between iCBT and face-to-face sessions)
**PTSD**					
	Turgoose et al (2018, veterans) [38]	Video-based exposure (n=10); video-based cognitive processing therapy (n=6); video-based CBT (n=5); mixed interventions (n=11); telephone mindfulness (n=1); video-based behavioral activation (n=2); video-based eye movement desensitization and reprocessing (n=1); video-based anger management (n=2); video-based general coping and psychoeducation interventions (n=3)	Face-to-face (n=41)	In total, 18 studies looked at the clinical effectiveness of teletherapy interventions. All of these studies reported that teletherapy was associated with significant reductions in PTSD symptoms, regardless of the type of intervention used, except one study that only measured anger in veterans with PTSD. Of those studies that used follow-up measures, all but one found these changes to be present at 3 or 6 months following treatment. In total, 67% (12/18) of studies compared teletherapy with in-person interventions. In all, 9 studies concluded that teletherapy was as effective as in-person therapy. Two suggested in-person therapy produced significantly greater reductions in PTSD symptoms (though neither were randomized), and 1 study found that teletherapy was more effective than in-person therapy.	No combined data available
	Olthuis et al (2016b) [36]	Video-based CBT (n=3); video-based cognitive processing therapy (n=3); internet CBT with therapist email or telephone support (n=9); video-based prolonged exposure (n=2); telephone mindfulness (n=1); video-based behavioral activation and exposure (n=1)	Face-to-face (n=8); internet-based supportive counseling (n=1); treatment as usual (n=2); telephone (n=1); self-help iCBT (n=1); waiting list (n=6)	Overall, telehealth interventions showed significant improvement in PTSD symptoms postintervention (n=18), at 3- to 6-month follow-up (n=11), and at 7- to 12-month follow-up (n=3); videoconferencing: in total, 9 studies examined videoconferencing interventions for PTSD. Results showed a significant improvement in PTSD symptoms at postintervention. There was no difference in improvements in PTSD symptoms between telehealth and face-to-face interventions at posttreatment (n=7); however, face-to-face interventions showed a significantly greater improvement at 3- to 6-month follow-up (n=5). Internet-based therapy delivered with telephone or email support: in total, 8 studies examined internet-delivered interventions with telephone or email support. Results showed significant improvements in PTSD symptoms at postintervention. Furthermore, telehealth interventions were found to show a significantly greater improvement in PTSD symptoms as compared with waitlist controls (n=6). There were no data comparing these interventions with face-to-face treatments. No follow-up data were available.	Total: within group: pre- and postintervention: g=0.81 (95% CI 0.65 to 0.97), n=18 (favors telehealth); preintervention to 3- to 6-month follow-up: g=0.78 (95% CI 0.59 to 0.97), n=11 (favors telehealth) preintervention to 7- to 12-month follow-up: g=0.75 (95% CI 0.25 to 1.26), n=3 (favors telehealth); Between group: as compared with waitlist control postintervention: g=0.6 (95% CI 0.51 to 0.86), n=6 (favors telehealth) compared with face-to-face treatment for PTSD postintervention: g=–0.05 (95% CI –0.31 to 0.20), n=7 (no difference) compared with face-to-face treatment for PTSD 3- to 6-month follow-up: g=–0.25 (95% CI –0.44 to –0.07), n=5 (favors face-to-face). Videoconferencing: within group: pre- and postintervention: g=0.71 (95% CI 0.47 to 0.96), n=8 (favors telehealth). Between group: compared with waitlist control postintervention: No data compared with face-to-face treatment for PTSD postintervention: g=–0.05 (95% CI –0.31 to 0.20), n=7 (no difference). Internet-delivered interventions with telephone or email support. Within group: pre- and postintervention: g=0.94 (95% CI 0.69 to 1.20), n=8 (favors telehealth). Between group: compared with waitlist control postintervention: g=0.73 (95% CI 0.56 to 0.91), n=5 (favors telehealth) compared with face-to-face treatment for PTSD postintervention (no data)
	Bolton and Dorstyn (2015) [24]	iCBT with therapist support via telephone calls, introductory face-to-face meetings, or emails (n=6); video-based CBT (n=5)	Face-to-face (n=5); supportive counseling (n=1); waitlist (n=1); no control (n=4)	Therapist-assisted internet programs. Statistically significant reductions in the severity of depression and anxiety symptoms (including PTSD) were associated with therapist-assisted internet programs in five studies, including significant large reductions in fear reactions, suicidal ideation, social functioning, and insomnia. Treatment effects 1-6 months posttelepsychology were mixed, with both deterioration and continued improvement found in psychological functioning. This included an increased risk of alcohol consumption over time but also a decline in PTSD and depression symptoms in participants using internet programs. Videoconferencing: video-based interventions also produced short-term reductions in affective symptoms; however, face-to-face therapy demonstrated slightly higher treatment gains. The longer-term effectiveness of videoconferencing was reported in only two studies which showed nonsignificant effect sizes at follow-up.	No useful synthesis of data
**Depression**					
	Harerimana et al (2019, older adults) [20]	Telephone-based (n=3); video-based (n=2); web based (n=1)	Waiting list (n=NR^h^) treatment as usual (n=NR)	Telephone: three studies examined a telephone-based intervention. One study found that a home electronic messaging service reduced emergency room and hospital visits. Another study found that older adult veterans given a combined telephone-based psychotherapy and long-term illness management intervention showed significant reductions in depression as compared with usual care. However, a third study found that adding telecoaching to a web intervention did not significantly improve symptoms compared with providing only the web intervention. Videoconferencing: two studies examined Skype-based videoconferencing interventions with inconsistent results. One study found that depression scores improved significantly from baseline but got worse at the 2-month follow-up. Another found that face-to-face and Skype-based intervention were not significantly different at postintervention and shorter follow-ups, but at 36 months, the telehealth intervention showed significantly larger improvements in symptoms. Web-based CBT: one web-based CBT intervention was effective in reducing depression symptoms (*P*=.04), even with high rates of attrition.	No combined data available
	Berryhill et al (2019a) [22]	Video-based CBT (n=12); video-based behavioral activation (n=5); video-based acceptance and behavioral therapy (n=1); video-based exposure (n=3); video-based metacognitive therapy (n=1); video-based problem-solving therapy (n=2); video-based therapy in multiple modalities (n=9)	Face-to-face psychotherapy (n=16); face-to-face or telephone (n=2); no control (n=15)	In total, 67% (22/33) of studies included reported statistically significant reductions in depressive symptoms following videoconference-based psychotherapy. Most controlled studies reported inconsistent results while comparing face-to-face and video-based psychotherapy.	No combined data available
	Coughtrey and Pistrang (2018) [26]	Telephone-based CBT (n=10)	Telephone emotion-focused therapy (n=1); treatment as usual (n=5); no control (n=4)	In total, 83% (5/6) of RCTs on depression reported a significant reduction in depression symptoms following telephone-delivered CBT (n=3) or IPT^i^ (n=2). These studies included people with recurrent depression (n=1), HIV (n=1), multiple sclerosis (n=1), and people from rural Latino communities (n=1). Two RCTs reported follow-up: only one of these found the maintenance of reductions in depressive symptoms. One RCT found that depression symptoms were not significantly reduced in veterans. One quasiexperimental study found significant reductions in depression following telephone-delivered CBT, with similar patterns of change found in the comparison group. Three uncontrolled studies reported statistically significant reductions in depression following telephone-delivered CBT, including people with Parkinson disease (n=1), HIV (n=1), and veterans with depression (n=1).	RCTs: Cohen *d* range: 0.25-1.98 (median 0.58), n=5; uncontrolled: Cohen *d* range: 1.13-1.90 (median 1.25), n=2
	Dorstyn et al (2013; minority ethnicity communities) [21]	Telephone CBT (n=2); telephone supportive counseling (n=1); telephone structural ecosystems therapy (n=1); internet-based CBT with weekly individual sessions (n=2); internet telepsychiatry (n=1); internet supportive counseling and personalized email correspondence (n=1)	Face-to-face (n=1); treatment as usual (n=3); minimal support control or waitlist (n=2); no control (n=2)	Telephone- and internet-mediated services were associated with significant improvements in the measurements of depression, anxiety, quality of life and psychosocial functioning. The review also found that two studies demonstrated similar effects on depression ratings (CES-D^j^) in telephone and face-to-face psychotherapy. Three studies reported longer-term effects of telecounseling, with conflicting findings.	No combined data available
Carers of people with dementia (for depressive symptoms)	Lins et al (2014) [31]	Telephone counseling (n=9)	Friendly calls (n=3); treatment as usual (n=6)	Telephone counseling without any additional intervention showed significant reductions in depressive symptoms in three studies; however, two additional studies showed no differences between groups. A study of telephone counseling with video sessions showed reductions in depressive symptoms in the intervention group but these did not significantly differ from the control group. One study found that telephone counseling with video sessions and a workbook showed significant reductions in depressive symptoms. Burden, distress, anxiety, quality of life, satisfaction, and social support outcomes were inconsistent. Results show that it is still unclear whether telephone counseling can reduce caregiver burden.	Telephone counseling only: depressive symptoms: n=3, SMD=0.32 (95% CI 0.01 to 0.63), *P*=.04; burden: n=4, SMD=0.45 (95% CI –0.01 to 0.90), *P=*.05
Substance use disorders	Lin et al (2019) [30]	Video or telephone-based psychotherapy (n=10); remote medication management (n=3; patient presents at local clinic with nurse and is connected to a physician at a distant site via videoconference)	Face-to-face psychotherapy (n=7); telephone (n=2); treatment as usual (n=1); no control (n=3)	Tobacco: videoconferencing interventions were not significantly better than in-person (n=1) or telephone (n=2) conditions in terms of abstinence. Alcohol: no significant difference in alcohol use outcomes as compared with usual treatment (n=1), but lower dropout was reported in the telemental health intervention (n=1). Opioid: no significant difference in abstinence between videoconference-based psychotherapy and in-person psychotherapy for methadone patients (n=2), and no difference in time to abstinence (n=1). Notably, none of the included studies described a noninferiority design that specifically assessed whether the intervention was not significantly worse than usual in-person delivered care. Overall, most studies suggested telemental health interventions were an effective alternative, especially when access to treatment is otherwise limited.	No combined data available
**Nonspecific**					
	Hassan and Sharif (2019, refugee populations) [29]	Not specified videoconferencing treatment intervention (n=2); video-based CBT (n=7); video-based psychoeducation (n=2); video-based relapse prevention (n=1); video-based treatment management (n=1); video-based evaluation of competency to stand trial (n=1)	Face-to-face (n=14)	Five studies compared remote and face-to-face interventions in symptom reduction. Two reviews found greater improvement in the remote intervention, whereas 3 found no significant difference between the intervention and control groups.	No combined data available
	Norwood et al (2018) [34]	Video-based CBT (n=10)	Face-to-face CBT (n=10)	All 10 studies showed that video-based CBT improved symptom severity. Eight studies offered follow-up data, and the postintervention improvement was maintained in all of them. Symptom reduction in video-based CBT was noninferior to face-to-face sessions across all six studies which offered a face-to-face comparison.	No combined data available
	Drago et al (2016) [27]	Videoconferencing (n=24)	Face-to-face (n=23). No comparator (n=1)	In total, 14 RCTs focused on efficacy of remote psychiatric counseling. There was no difference between treatment outcomes in remote and face-to-face settings.	Videoconferencing versus face-to-face therapy: SMD=–0.11 (95% CI –0.41 to 0.18)
	Garcia-Lizana and Munoiz-Mayorga (2010) [28]	Videoconferencing for diagnosis and follow-up (n=3); video-based evaluation of competency to stand trial (n=1); nonspecific video-based CBT (n=5); video-based psychoeducation and counseling (n=1)	Face-to-face (n=10)	Across seven studies, there was no statistically significant difference between telepsychiatry and face-to-face interventions in symptom reduction. Across three studies, there was no statistically significant difference between telepsychiatry in improvements in quality of life.	No combined data available

^a^CBT: cognitive behavioral therapy.

^b^ACT: acceptance and commitment therapy.

^c^RCTs: randomized controlled trials.

^d^OCD: obsessive-compulsive disorder.

^e^PTSD: posttraumatic stress disorder.

^f^iCBT: internet-based cognitive behavioral therapy.

^g^SMD: standardized mean difference.

^h^NR: not reported.

^i^IPT: interpersonal psychotherapy.

^j^CES-D: Center for Epidemiological Studies-Depression Scale.

### Implementation Outcomes

#### Overview

Implementation outcomes were reported in eight reviews [21,24,25,27,28,30,32,38]. Relevant outcomes included assessment comparability (n=2 reviews), fidelity to intervention and competence of therapists (n=1) [38], patient adherence to intervention (n=3) [21,24,28], patient attendance (n=4) [21,25,30,38], safety (n=2) [24,38], and technical difficulties (n=3) [24,25,38].

#### Assessment Comparability

Limited evidence from one review suggests that videoconferencing can be used to conduct assessment, which is consistent with face-to-face assessment, with a correlation coefficient of 0.73 (95% CI 0.63-0.83) between the conclusions of videoconference and face-to-face assessments [27]. A review of telephone assessments found lack of properly performed studies on telephone assessments [32].

#### Fidelity and Competence of Therapists

One review [38] found that fidelity and therapist competence using telemental health had been found to be comparable with face-to-face interventions in 3 studies of interventions for PTSD in veterans.

#### Patient Adherence to Intervention

From 3 reviews [21,24,28] examining patients’ adherence to remote interventions the general consensus was that the comprehension of tasks and completion rates are high during both telephone and video-based CBT. However, another review reported mixed findings, with one of the two included relevant studies reporting better adherence in the face-to-face intervention group for patients with PTSD, whereas another study on patients with depression reported equivalent adherence in remote and face-to-face conditions [28].

#### Patient Attendance

An increase in uptake and access to care following the introduction of telemental health has been reported in the reviews of depression treatment in older adults [20], PTSD treatment in veterans [38], and substance use disorder treatment [30]. Dropouts tended to be comparable with face-to-face interventions [30,38]. However, one review included a study reporting difficulty in reaching ethnic minority patients with depression [21].

#### Safety

Patient safety while using remote interventions has been reported only in the reviews of populations with PTSD. Two reviews agreed that safety was acceptable, with one reporting that with correct steps taken, safety could usually be managed in remote settings [38], and another study reported that client safety was deemed satisfactory (however, no further details were provided on this result) [24].

#### Technical Difficulties

A total of 3 reviews reported technical difficulties, none of which were identified as severe barriers to the implementation of remote technology. A review of older adults with depression found that four studies reported mistrust in technology [25], while the challenges of a more logistical nature, such as low image resolution and connectivity problems, were reported in a review of video-based PTSD interventions for veterans [38]. Another review reported findings from one included study that participants preferred mobile apps to supplement remotely delivered support [24]. Table 3 presents further details on the implementation outcomes.

**Table 3:** Implementation outcomes.

Outcome	Study	Assessment or treatment	Main diagnosis	Intervention (number of studies)	Comparator (number of studies)	Results	
**Assessment comparability**							
	Drago et al (2016) [27]	Assessment and treatment	Multiple	Videoconferencing (n=24)	Face-to-face (n=23); no comparator (n=1)	Assessment was found to be highly consistent between remote and face-to-face settings; correlation coefficient=0.73 (95% CI 0.63-0.83).	
	Muskens et al (2014) [32]	Assessment	Multiple	Telephone diagnostic interviewing (n=16)	Face-to-face diagnostic interviewing (n=16)	There were a few studies that were properly performed to draw conclusions regarding the comparability of telephone and face-to-face interviews for psychiatric morbidity. Telephone interviewing for research purposes in depression and anxiety may however be a valid method.	
Fidelity and competence of therapists	Turgoose et al (2018, veterans) [38]	Treatment	PTSD^a^	Video-based exposure (n=10); video-based cognitive processing therapy (n=6); video-based CBT (n=5); mixed interventions (n=11); telephone mindfulness (n=1); video-based behavioral activation (n=2); video-based eye movement desensitization and reprocessing (n=1); video-based anger management (n=2); video-based general coping and psychoeducation interventions (n=3)	Face-to-face (n=41)	High levels of fidelity and therapist competence (n=3), with no significant differences compared with face-to-face settings.	
**Patient adherence to intervention**							
	Bolton and Dorstyn (2015) [24]	Treatment	PTSD	Internet-based CBT^b^ with therapist support via telephone calls, introductory face-to-face meetings, or emails (n=6); video-based CBT (n=5)	Face-to-face (n=5); supportive counseling (n=1); waitlist (n=1); no control (n=4)	Qualitative feedback revealed that the comprehension of the therapy materials was high, with participants completing a set of homework tasks (n=5).	
	Dorstyn et al (2013, ethnic minorities) [21]	Treatment	Depression	Telephone CBT (n=2); telephone supportive counseling (n=1); telephone structural ecosystems therapy (n=1); internet-based CBT with weekly individual sessions (n=2); internet telepsychiatry (n=1); internet supportive counseling and personalized email correspondence (n=1)	Face-to-face (n=1); treatment as usual (n=3); minimal support control or waitlist (n=2); no control (n=2)	Most studies reported good treatment adherence with rates of completion of 75-97%.	
	Garcia-Lizana and Munoiz-Mayorga (2010) [28]	Assessment and treatment	Multiple	Videoconferencing for diagnosis and follow-up (n=3); video-based evaluation of competency to stand trial (n=1); nonspecific video-based CBT (n=5); video-based psychoeducation and counseling (n=1)	Face-to-face (n=10)	Across two studies, mixed results were found for treatment adherence, with one study finding no difference and another reporting better adherence in the face-to-face group.	
**Patient attendance**							
	Dorstyn et al (2013, ethnic minorities) [21]	Treatment	Depression	Telephone CBT (n=2); telephone supportive counseling (n=1); telephone structural ecosystems therapy (n=1); internet-based CBT with weekly individual sessions (n=2); internet telepsychiatry (n=1); internet supportive counseling and personalized email correspondence (n=1)	Face-to-face (n=1); treatment as usual (n=3); minimal support control or waitlist (n=2); no control (n=2)	One study reported difficulty reaching participants by telephone resulting in fewer sessions completed.	
	Christensen et al (2019, older adults) [25]	Treatment	Depression or a range of diagnoses including depression	Video consultations for telepsychiatry (n=21)	Face-to-face (11), no control (10)	Video consultations increased access to care and removed barriers such as having to travel (n=4).	
	Lin et al (2019) [30]	Treatment	Substance use disorders	Video- or telephone-based psychotherapy (n=10); remote medication management (n=3; patient presents at local clinic with nurse and are connected to a physician at a distant site via videoconference)	Face-to-face psychotherapy (n=7); telephone (n=2); treatment as usual (n=1); no control (n=3)	Most studies reported increased retention in telemental health groups (n=4); however, no difference in in number of sessions attended was sometimes reported (n=2). One alcohol addiction study reported lower dropout in the telemental health group, and more patients in this group were still in treatment at 6 months and one year. Two opioid addiction studies found that videoconference interventions resulted in a better retention of participants up to one year as compared with those receiving in-person care. Another opioid study found >50% retention at 12 weeks but did not have a comparison group. However, another two studies found no difference between videoconference-delivered psychotherapy and in-person psychotherapy in the number of sessions attended.	
	Turgoose et al (2018 veterans) [38]	Treatment	PTSD	Video-based exposure (n=10); video-based cognitive processing therapy (n=6); video-based CBT (n=5); mixed interventions (n=11); telephone mindfulness (n=1); video-based behavioral activation (n=2); video-based eye movement desensitization and reprocessing (n=1); video-based anger management (n=2); video-based general coping and psychoeducation interventions (n=3)	Face-to-face (n=41)	In the majority of cases, there were no differences between teletherapy and in-person treatments on dropout or attendance. There was some indication that teletherapy may help to increase uptake.	
**Safety**							
	Bolton and Dorstyn (2015) [24]	Treatment	PTSD	Internet-based CBT with therapist support via telephone calls, introductory face-to-face meetings, or emails (n=6); video-based CBT (n=5)	Face-to-face (n=5); supportive counseling (n=1); waitlist (n=1); no control (n=4)	Client safety was deemed satisfactory.	
	Turgoose et al (2018, veterans) [38]	Treatment	PTSD	Video-based exposure (n=10); video-based cognitive processing therapy (n=6); video-based CBT (n=5); mixed interventions (n=11); telephone mindfulness (n=1); video-based behavioral activation (n=2); video-based eye movement desensitization and reprocessing (n=1); video-based anger management (n=2); video-based general coping and psychoeducation interventions (n=3)	Face-to-face (n=41)	There might be some occasions where veterans have concerns about exposure tasks due to the lack of physical presence of the therapist; however, overall, it was established that these can be used just as effectively remotely. If appropriate steps are taken to manage safety, then episodes of acute suicidality can also be managed.	
**Technical difficulties**							
	Bolton and Dorstyn (2015) [24]	Treatment	PTSD	Internet-based CBT with therapist support via telephone calls, introductory face-to-face meetings, or emails (n=6); video-based CBT (n=5)	Face-to-face (n=5); supportive counseling (n=1); waitlist (n=1); no control (n=4)	Minimal technical difficulties were encountered (n=1); participants reported that they would have preferred different forms of media, for example, a mobile app, to supplement support (n=1).	
	Christensen et al (2019, older adults) [25]	Treatment	Depression or a range of diagnoses including depression	Video consultations for telepsychiatry (n=21)	Face-to-face (11), no control (10)	Challenges such as mistrust in technology were reported frequently (n=4).	
	Turgoose et al (2018, veterans) [38]	Treatment	PTSD	Video-based exposure (n=10); video-based cognitive processing therapy (n=6); video-based CBT (n=5); mixed interventions (n=11); telephone mindfulness (n=1); video-based behavioral activation (n=2); video-based eye movement desensitization and reprocessing (n=1); video-based anger management (n=2); video-based general coping and psychoeducation interventions (n=3)	Face-to-face (n=41)	Commonly reported technical difficulties were low-image resolution on videoconferencing technology, not being able to connect, and audio delays.	

^a^PTSD: posttraumatic stress disorder.

^b^CBT: cognitive behavioral therapy.

### Acceptability Outcomes

#### Overview

Acceptability outcomes were reported in 10 reviews [20,21,24,25,28-31,34,38]. Relevant outcomes included clinician satisfaction (n=5) [20,28,29,31,38], therapeutic alliance (n=6) [24,25,30,31,34,38], patient satisfaction (n=7) [21,25,28-31,38], and convenience (n=3) [25,30,31].

#### Clinician Satisfaction

Overall, clinicians tend to report a preference for face-to-face interventions for both assessment and treatment [28,29]. However, some reviews have reported that clinicians find video-based therapies to be acceptable [29,38]. One review of remote interventions for the carers of people with dementia found that counselors felt they might need more support via debriefing following remote counseling sessions. They also reported problems when the reactions of carers could not be ascertained via remote technology along with the feelings of helplessness due to the impersonal nature of remote technology [31]. Health care providers using remote interventions in older adults noted the practical benefits of telehealth [20].

#### Therapeutic Alliance

Overall, therapeutic alliances were reported to be comparable with face-to-face interventions. However, some patient groups were found to feel more comfortable talking to therapists face to face, if possible, such as older female adults [25] or veterans [38]. A meta-analysis was conducted in one review, which found that although standardized mean differences in alliance ratings were not significantly different, the lower limit of the 95% CI fell outside the prespecified limit of noninferiority, indicating that videoconference interventions may be inferior to face-to-face treatment, likely the result of therapist-rated (but not patient-rated) alliance scores being lower in the videoconference groups [34].

#### Patient Satisfaction

High patient satisfaction was generally reported across seven reviews, and patients tended to find remote interventions as satisfactory as face-to-face alternatives. This was true in substance use disorders [30], depression [21,25,28,29], PTSD [38], older adults [25], ethnic minorities [21], and carers of patient populations with dementia [31]; however, Hassan and Sharif [29] reported a few studies indicating preference for face-to-face interventions. A review of older people noted that initial skepticism between both service users and providers tended to dissipate following positive experiences of videoconferencing; moreover, with appropriate support and access to technology, patients who had not previously used computers reported positive experiences of video calls [25]. Accepting the need for treatment to be in teletherapy form instead of face-to-face therapy was reported as important in a study of veterans with PTSD [38].

#### Convenience

Patients reported the benefits of added convenience of therapy sessions at home via remote interventions for both depression [25,31] and substance use disorders [30]. Table 4 presents further details on the acceptability outcomes.

**Table 4:** Acceptability outcomes.

Outcome	Study	Assessment or treatment	Main diagnosis	Intervention (number of studies)	Comparator (number of studies)	Results	
**Clinician satisfaction**							
	Garcia-Lizana and Munoiz-Mayorga (2010) [28]	Assessment and treatment	Multiple	Videoconferencing for diagnosis and follow-up (n=3); video-based evaluation of competency to stand trial (n=1); nonspecific video-based CBT^a^ (n=5); video-based psychoeducation and counseling (n=1)	Face-to-face (n=10)	The lowest level of satisfaction was found to be in the videoconferencing group in two studies that examined clinician satisfaction.	
	Hassan and Sharif (2019; refugee populations) [29]	Assessment and treatment	Multiple	Not specified videoconferencing treatment intervention (n=2); video-based CBT (n=7); video-based psychoeducation (n=2); video-based relapse prevention (n=1); video-based treatment management (n=1); video-based evaluation of competency to stand trial (n=1)	Face-to-face (n=14)	Clinicians tended to report higher satisfaction in the face-to-face interventions; however, most reported good satisfaction with the videoconference group.	
	Turgoose et al (2018; veterans) [38]	Treatment	PTSD^b^	Video-based exposure (n=10); video-based cognitive processing therapy (n=6); video-based CBT (n=5); mixed interventions (n=11); telephone mindfulness (n=1); video-based behavioral activation (n=2); video-based eye movement desensitization and reprocessing (n=1); video-based anger management (n=2); video-based general coping and psychoeducation interventions (n=3)	Face-to-face (n=41)	One study reported that clinicians delivering therapy found teletherapy acceptable, with no difference with in-person therapies.	
	Harerimana et al (2019; older adults) [20]	Treatment	Depression	Telephone based (n=6); video based (n=2); web based (n=1)	Waiting list (NR^c^); treatment as usual (NR)	Health care providers have positive perceptions and notice practical benefits associated with the use of telehealth for delivery of community mental health care (n=1). However, nurses of a telepsychiatry consultation generally did not rate it positively (n=1).	
	Lins et al (2014) [31]	Support for carers of people with dementia (depressive symptoms)	Carers of people with dementia (for depressive symptoms)	Telephone counseling (n=9, n=2 reporting implementation outcomes)	Friendly calls (n=3); treatment as usual (n=6)	Spatial distance could be a problem because counselors cannot see the reactions of carers (n=1). Counselors also expressed a need for a debriefing with colleagues after counseling sessions.	
**Therapeutic alliance**							
	Bolton and Dorstyn (2015) [24]	Treatment	PTSD	Internet-based CBT with therapist support via telephone calls, introductory face-to-face meetings, or emails (n=6); video-based CBT (n=5)	Face-to-face (n=5); supportive counseling (n=1); waitlist (n=1); no control (n=4)	Good therapeutic alliance reported (n=5)	
	Christensen et al (2019, older adults) [25]	Treatment	Depression or a range of diagnoses including depression	Video consultations for telepsychiatry (n=21)	Face-to-face (11), no control (10)	Video sessions were considered better than telephone sessions because of their similarity to face-to-face sessions (n=2); however, in one study, female patients found videoconferencing interventions more impersonal than face-to-face sessions. One clinician reported reduced communication intensity because of less clear facial movements (n=1).	
	Lin et al (2019) [30]	Treatment	Substance use disorders	Video- or telephone-based psychotherapy (n=10); remote medication management (n=3; patient presents at local clinic with nurse and are connected to a physician at a distant site via videoconference)	Face-to-face psychotherapy (n=7); telephone (n=2); treatment as usual (n=1); no control (n=3)	Participant and therapist ratings of therapeutic alliance ratings were high in both videoconference and in-person interventions (n=1).	
	Lins et al (2014) [31]	Support for carers of people with dementia (depressive symptoms)	Carers of people with dementia (for depressive symptoms)	Telephone counseling (n=2 reporting implementation outcomes)	Friendly calls (n=3); treatment as usual (n=6)	Counselors can feel frustrated and helpless during telephone counseling because it is relatively impersonal (n=1).	
	Norwood et al (2018) [34]	Treatment	Multiple	Video-based CBT (n=10)	Face-to-face CBT (n=10)	Six studies used a face-to-face condition as a control group, with four finding that therapeutic alliance was noninferior in the videoconferencing condition as compared with face-to-face conditions. The remaining two reported that alliance was higher in the face-to-face group, though one reported no difference in participant rated alliance, only significantly higher therapist-rated alliance for the face-to-face group. Standardized mean difference in alliance ratings –0.30 (95% CI –0.67 to 0.07), *P=*.11, n=4. The lower limit of the 95% CI fell outside the prespecified limit of noninferiority (Cohen *d*=−0.50), indicating that with respect to working alliance, videoconference interventions were inferior to face-to-face treatment.	
	Turgoose et al (2018, veterans) [38]	Treatment	PTSD	Video-based exposure (n=10); video-based cognitive processing therapy (n=6); video-based CBT (n=5); mixed interventions (n=11); telephone mindfulness (n=1); video-based behavioral activation (n=2); video-based eye movement desensitization and reprocessing (n=1); video-based anger management (n=2); video-based general coping and psychoeducation interventions (n=3)	Face-to-face (n=41)	Although most studies found that alliance was equivalent in teletherapy and in-person conditions, some suggested that veterans may feel more comfortable talking to therapists face-to-face. Challenges in detecting body language were reported, but overall clinicians felt that teletherapy did not affect their ability to establish rapport.	
**Patient satisfaction**							
	Christensen et al (2019; older adults) [25]	Treatment	Depression or a range of diagnoses including depression	Video consultations for telepsychiatry (n=21)	Face-to-face (11), no control (10)	High levels of patient satisfaction and acceptability were frequently reported, and there were no significant differences between face-to-face and videoconferencing in RCT^d^ studies. Patients preferred the reduced waiting time (n=1). Some patients reported initial skepticism as a reason for preference of face-to-face interventions, however this usually dissipated with use of remote technology.	
	Dorstyn et al (2013, ethnic minorities) [21]	Treatment	Depression	Telephone CBT (n=2); telephone supportive counseling (n=1); telephone structural ecosystems therapy (n=1); internet-based CBT with weekly individual sessions (n=2); internet telepsychiatry (n=1); internet supportive counseling and personalized email correspondence (n=1)	Face-to-face (n=1); treatment as usual (n=3); minimal support control or waitlist (n=2); no control (n=2)	Consistent patient satisfaction was reported.	
	Garcia-Lizana (2010) [28]	Assessment and treatment	Multiple	Videoconferencing for diagnosis and follow-up (n=3); video-based evaluation of competency to stand trial (n=1); nonspecific video-based CBT (n=5); video-based psychoeducation and counseling (n=1)	Face-to-face (n=10)	Patients generally appeared satisfied with the technology utilized and its quality (n=2). High satisfaction was reported in other studies; however, it is unclear if satisfaction was generated by the program or the technology (n=5).	
	Hassan and Sharif (2019; refugee populations) [29]	Assessment and treatment	Multiple	Not specified videoconferencing treatment intervention (n=2); video-based CBT (n=7); video-based psychoeducation (n=2); video-based relapse prevention (n=1); video-based treatment management (n=1); video-based evaluation of competency to stand trial (n=1)	Face-to-face (n=14)	Most studies reported a high satisfaction with videoconference interventions (n=3) or no difference in satisfaction as compared with face-to-face groups (n=3); however, one study reported a lower satisfaction as compared with face-to-face sessions.	
	Lin et al (2019) [30]	Treatment	Substance use disorders	Video- or telephone-based psychotherapy (n=10); remote medication management (n=3; patient presents at local clinic with nurse and are connected to a physician at a distant site via videoconference)	Face-to-face psychotherapy (n=7); telephone (n=2); treatment as usual (n=1); no control (n=3)	Satisfaction was generally quite high in videoconference interventions and that participants would recommend the intervention to others.	
	Lins et al (2014) [31]	Support for carers of people with dementia (depressive symptoms)	Carers of people with dementia (for depressive symptoms)	Telephone counseling (n=9, n=2 reporting implementation outcomes)	Friendly calls (n=3), treatment as usual (n=6)	Reservations expressed about getting advice from an unknown person (n=1). Both studies reported that carers found the information given helpful and were grateful for it. One study found that telephone counseling helped alleviate loneliness in carers.	
	Turgoose et al (2018; veterans) [38]	Treatment	PTSD	Video-based exposure (n=10); video-based cognitive processing therapy (n=6); video-based CBT (n=5); mixed interventions (n=11); telephone mindfulness (n=1); video-based behavioral activation (n=2); video-based eye movement desensitization and reprocessing (n=1); video-based anger management (n=2); video-based general coping and psychoeducation interventions (n=3)	Face-to-face (n=41)	Patients found teletherapy and face-to-face treatments equally satisfactory: accepting the need for treatments to be in teletherapy form was shown to be important.	
**Convenience**							
	Christensen et al (2019; older adults) [25]	Treatment	Depression or a range of diagnoses including depression	Video consultations for telepsychiatry (n=21)	Face-to-face (11), no control (10)	Patients reported that video consultations were more relaxing, and it was convenient to stay at home (n=3).	
	Lin et al (2019) [30]	Treatment	Substance use disorders	Video or telephone-based Psychotherapy (n=10) remote medication management (n=3; patient presents at local clinic with nurse and are connected to a physician at a distant site via videoconference)	Face-to-face psychotherapy (n=7); telephone (n=2); treatment as usual (n=1); no control (n=3)	Participants found the increased convenience important as they would have had difficulty obtaining the intervention without telemental health (n=1).	
	Lins et al (2014) [31]	Support for carers of people with dementia (depressive symptoms)	Carers of people with dementia (for depressive symptoms)	Telephone counseling (n=9, n=2 reporting implementation outcomes)	Friendly calls (n=3); treatment as usual (n=6)	Carers found telephone counseling good because it avoided the stress involved in coordinating an appointment (n=1). Needs for 24-hour counselor availability (n=1).	

^a^CBT: cognitive behavioral therapy.

^b^PTSD: posttraumatic stress disorder.

^c^NR: not recorded.

^d^RCT: randomized controlled trial.

### Cost-Effectiveness

A total of 2 reviews presented conclusions regarding the economic impact of telepsychiatry [29,33]. One review concluded that telepsychiatry can be cost-effective as compared with face-to-face interventions, particularly in rural areas where there were lower numbers of consultations required before telepsychiatry becomes more cost-effective (combatting initial equipment costs) [29]. The second review, in which the focus was on the cost-effectiveness of telepsychiatry, reported that 60% (15/25) of the included studies found telepsychiatry programs to be less expensive than standard in-person care, due to savings such as the travel time and reduced need for patients and their families to take time off work. However, the remaining studies included in the review concluded that telepsychiatry programs were more expensive, particularly because of videoconferencing equipment costs (n=8) or found no difference in costs (n=1). The review also found a large range in reported costs, with, for example, a long-term delivery of telepsychiatry for veterans ranging from US $930 to US $2116 per patient. The cost-effectiveness analyses within the review suggested that telepsychiatry was less cost-effective than face-to-face alternatives. Accordingly, the review concluded that variation was due to a large disparity in the reporting of costs, for example, whether personnel costs or initial equipment costs were included, and that there remains a need for future efforts to determine the cost-effectiveness of different forms of telepsychiatry, particularly for different disorders and applications of remote technology (eg, consultation vs therapy). In addition, a third review [21] examined health service utilization, which impacts cost-effectiveness. They found that the rates of antidepressant and health service utilization were similar in the 3 months following both telephone and web-based counseling as compared with usual care or face-to-face controls.

### Guidelines

Only one review [37] of guidelines for telemental health met the inclusion criteria. This review comprehensively summarizes the guidance published to date, including guidance on decisions about the appropriateness of e–mental health, ensuring the competence of mental health professionals, legal and regulatory issues, confidentiality, professional boundaries, and crisis intervention. Recommendations from 19 guidelines were characterized as either firm (50% or more of the guidelines recommended this) or tentative (fewer than 50% of the guidelines recommended this). The review identified the following as firm recommendations: ensuring that the remote interventions were appropriate for the needs of individual patients and within the boundaries of therapist competence, as well as adhering to laws and regulations; maintaining confidentiality and seeking informed consent (including for specific aspects of remote appointments such as data security); and ensuring that geographically accessible in-person clinical support is available in the case of a crisis or emergency. Guidelines suggested a higher risk of harm for people with cognitive impairments and psychotic disorders but did not provide stronger recommendations on how to adapt delivery to these populations. Furthermore, a minority of guidelines discussed remote technology in young people, with the main message being the importance of checking consent with both the patient and parent. A full summary of the recommendations from the review can be found in Multimedia Appendix 5.

## Discussion

### Principal Findings

Our umbrella review retrieved a variety of recent relevant systematic reviews on which the future planning of telemental health implementation can be usefully drawn. Across the 19 reviews included in this umbrella review, the results suggest that the remote forms of assessment and intervention can produce at least moderate decreases in symptom severity for people affected by a variety of mental health conditions. Arguments are strongest for videoconferencing interventions, with multiple reviews concluding that outcomes appear comparable with face-to-face interventions at the end of treatment. However, at present, conclusions regarding longer-term results remain uncertain, whereas some reviews have reported the maintenance of positive effects up to six months for both videoconference- and telephone-based interventions, other reviews have suggested that effects are less long-lasting than for face-to-face interventions and the amount of evidence on which to base this assessment is limited. An avenue for future research could be to further examine possible differences between settings in which longer-term benefits have been found compared with those which did not find this.

Reviews also suggest that remote interventions are acceptable to service users participating in studies who tend to report being as satisfied as in face-to-face interventions. This, along with reports that participants adhere to telemental health-based interventions at similar rates to face-to-face interventions, is promising with respect to adaptations during the COVID-19 crisis and for the future, but the reviews tend to relate to small-scale and carefully planned implementations of telemental health with volunteer participants, rather than to large-scale emergency implementations, as in the current crisis. Conclusions drawn from the reviews regarding specific factors that could impact patient satisfaction should also be noted. For example, a report of reduced skepticism following positive experiences [25], if confirmed, suggests that induction sessions and other methods of familiarizing patients with technology and making it accessible to them may improve acceptability and uptake. Clinician satisfaction varied more with reviews tending to conclude that although remote interventions may be acceptable, clinicians usually prefer face-to-face interventions. This may be related to the reports in some reviews that the clinician ratings of therapeutic alliance are poorer with telemental health [34,277]. Despite this, patients tend to feel that the alliance is on par with face-to-face interventions [30,34,38]. There is some suggestion that training and more experience with video and telephone-based technology for intervention delivery may alleviate this concern among therapists [277]; however, staff reports following an increased uptake in the COVID crisis appear to suggest continued concerns about rapport [2]. In the future, a thorough exploration of the exact reasons for acceptability and adherence would benefit the evidence base, for example, certain contextual factors such as appointment type, mood, and time of day may have a substantial influence on patient satisfaction and should be explored further.

Evidence yielded by reviews on the important questions of whether assessments appeared accurate and comprehensive and whether treatment was delivered as intended was limited. A total of 2 reviews examined the comparability of remote versus face-to-face assessment, with one review finding a good correlation between assessments and another finding that there was insufficient high-quality evidence published thus far to draw accurate and meaningful conclusions [27,32]. Regarding fidelity, we found one review that reported good therapist fidelity and competence in remotely delivered interventions in the context of service delivery for veterans with PTSD [38], but systematic reviews do not appear to yield evidence as to whether high fidelity and quality is achieved with telemental health interventions. Standardized training rooted in evidence will be important in ensuring high-quality intervention delivery and overcoming self-doubt among clinicians in delivering remote interventions [37,277,278].

A crucial question regarding the rapid adoption of remote technologies during the pandemic has been whether service users may drop out of or be excluded from care as a result. A minority of the reviews included relevant data, most of which were relatively reassuring. Reviews reported that remote interventions were convenient and that examining uptake reported an increase. When examined, retention was also comparable with face-to-face treatment [30,38]. Reports of technological difficulties were reassuringly few across reviews, although this may be more easily achieved with well-planned, smaller-scale implementations of telemental health that characterize research studies than with larger-scale implementation. However, one aspect of remote delivery on which reviews did not generally report is the risk of complete digital exclusion for patients who may not have the skills or resources to engage with remote therapy or assessments [1,2]. The implementation of telemental health across service systems is only likely to be beneficial if there are clear plans to prevent patients with limited access to technology from being at a disadvantage [279,280], whether by supporting them to engage with remote care or ensuring that equivalent care is available face-to-face.

Digital exclusion may result in the exacerbation of existing inequalities where already disadvantaged groups, such as older adults, people with sensory or cognitive impairment, or members of some BAME groups are at a greater risk of exclusion [1,9,281]. Some reviews have examined this issue [20,25]. A single review by Dorstyn et al [21] reported that members of predominantly North American ethnic minority communities with depression benefited from telecounseling. A more substantial evidence base is thus urgently required to evaluate the risk of exacerbating ethnic inequalities in mental health care access through telemental health adoption. Furthermore, many have argued that the shift to remote working may exclude older adults [25,281]. On the basis of one review [20] suggesting that videoconferencing interventions can be comparable with face-to-face sessions, and another review [25] finding high levels of patient satisfaction, therapeutic alliance, attendance, and convenience, this review suggests that effective remote intervention delivery may be feasible for older adults. This is encouraging as staying at home and avoiding infection during the pandemic is especially desirable for older adults. No reviews were found regarding other subgroups of potential concern, such as people with sensory or cognitive impairments, children and adolescents and their families, or people with comorbid mental and physical health conditions. We also did not find substantial evidence on settings of particular interest, such as mental health inpatient services (including the use of telemental health in compulsory detention processes) and crisis services.

### Limitations

The findings of this umbrella review should be considered along with a number of limitations. First, umbrella reviews aim to present an overview of findings from systematic reviews [282], making conclusions reliant on the quality and accuracy of reporting of included reviews and necessarily resulting in some loss of nuance when findings are pooled. For example, information regarding follow-up periods was not always clear in the conclusions presented in reviews, making a thorough examination of the longevity of benefits difficult. Although we included only reviews considered to be systematic (defined here as searching at least three databases and conducting a quality assessment when synthesizing quantitative data), it was apparent from our quality assessment that the majority of reviews lacked several attributes or characteristics of a high-quality review with robust conclusions, such as prespecified protocols and duplicate study selection. However, our aim was to gain a rapid overview, especially relevant to the current and future rapid implementation of telemental health, of the extent of supporting evidence available in previous literature regarding telemental health: the umbrella review method provides a useful route to achieving this. The inclusion of systematic reviews focused on methods other than randomized controlled trials and on guidance further increases the methodological variability of included reviews and studies, but it is a choice we have made to maximize the retrieval of material from which real-world important lessons can be learned regarding feasibility, acceptability, and implementation barriers and facilitators [283].

This review also aimed to summarize the outcomes related to the cost-effectiveness of remote delivery. We found only two reviews that summarized this outcome and only one that did so comprehensively. It is important that further work should be done to establish the cost-effectiveness of different forms of telemental health, for different patient groups, but there is a significant gap in the literature, despite efficiency being presented as one of the arguments made to support remote interventions [284].

Finally, this review aimed to summarize the literature published before the COVID-19 pandemic to identify evidence relevant to the current context and the recovery from the pandemic. However, the pandemic has given rise to a much more extensive switch to telemental health than previously, meaning that not all conclusions may be generalized to *the new normal*. In particular, the evidence retrieved in this review tends not to relate to the implementation of telemental health across whole catchment areas and does not yield much evidence relevant to currently highly salient issues such as risks of digital exclusion or exacerbation of mental health inequalities and economic disadvantage, which may well be exacerbated as a result of COVID-19 [1,2]. The conclusions of this review should be supplemented with the further scrutiny of the adoption of telemental health within the context of these societal changes, for example through discussion with people with lived experience (Textbox 1).

**Lived experience commentary.**

**Lived Experience Commentary (by SM From South London Applied Research Collaboration)**

As a somewhat long-term user of remote working to access assessment and treatment for a mental disorder, I found it fascinating to read this umbrella review. My experience of remote working has unsurprisingly arisen out of the spatial distancing restrictions imposed upon us all as a result of the SARS-CoV2 outbreak. No surprises there!

My personal experience has been, that although remote working is better than nothing, it isn’t in general as good as face-to-face working. Through my patient and public involvement work within my local Trust, I am aware that many fellow patients and carers are also of this view; remote working is okay as an interim measure, but could never replace the therapeutic alliance that can be achieved through sharing space as well as thoughts, feelings, and experiences.

I found it very interesting that the umbrella review found that, in general, clinicians have concerns about remote working as the vast majority of clinicians who I have liaised with via nonlocal advisory groups and PPI have voiced the opinion that they prefer assessing and delivering talking therapy remotely as they find it quicker and more efficient and means they don’t have to waste time traveling to different venues. This is understandable; however, for patients who have never met a clinical assessor or therapist before, it can be somewhat alienating to consult with a voice over a phone or an image on a smartphone rather than a present living breathing human being.

One of the main questions which came to mind as I read through the umbrella review was whether it was clarified in any of the individual reviews what service users meant or interpreted remote interventions being satisfactory or acceptable; better than nothing, okay, fine, what? It would have been useful to clarify this.

Another interesting point was that “reports of technological difficulties were reassuringly few across reviews.” Maybe it’s just me, but I have experienced regular and sometimes quite disastrous “technological difficulties,” being locked out of virtual meetings, poor audio and visual reception, loss of signal, not being able to access Wi-Fi, etc. This isn’t just annoying but can be incredibly frustrating if you are relying on a therapy session to help you to manage difficult matters in your life, including the mass COVID-19 related isolation and loneliness.

Regarding guidelines for remote working, I am glad that the umbrella review highlighted the gap in guideline provision for remote working with young people. A child psychiatrist I know well has found it very frustrating to have to rely on remote means of assessing young patients and has deep concerns about various risk and safety issues.

Overall, I think the umbrella review raises many pertinent questions and issues, and I hope that at some time in the future, there will be another review of the research literature that will begin in time to proliferate with regard to peoples’ experiences of using remote mental health assessments and interventions during this time of COVID-19.

### Conclusions

The research across a range of mental health conditions suggests that telemental health is potentially an effective, feasible, and acceptable tool for providing mental health treatment, at least when interventions are relatively well-designed and well-planned, as has been the case in research studies. Comparability in terms of symptom improvement and satisfaction relative to face-to-face methods suggests the move to telemental health to sustain mental health services during the pandemic has been a reasonable one; however, the context of this emergency implementation has been very different from most research studies. Further research should seek to build on existing evidence for establishing the long-term effectiveness and cost-effectiveness of telemental health in a range of groups and settings, such as including children and young people and inpatient acute services and focusing on issues of inclusion and reach. A further question on which further evidence would be highly desirable is the extent to which digital exclusion can be remedied, including the examination of interventions designed to include those with limited previous digital resources or skills. Future planning for telemental health implementation should draw on previous research evidence, often acquired in relatively small-scale studies, and on experiences of trying to engage large service user populations and most of the mental health workforce with remote technology delivery during the COVID-19 pandemic.

## Appendix

Multimedia Appendix 1 Search strategy.

Multimedia Appendix 2 Study overlap.

Multimedia Appendix 3 Included studies in reviews.

Multimedia Appendix 4 Detailed quality assessment.

Multimedia Appendix 5 Guideline recommendations.

## Abbreviations

AMSTAR: Assessment of Multiple Systematic Reviews
ARC: Applied Research Collaboration
BAME: Black, Asian, and minority ethnic
NHS: National Health Service
NIHR: National Institute for Health Research
PRISMA: Preferred Reporting Items for Systematic Reviews and Meta-Analyses
PTSD: posttraumatic stress disorder
