# Supplementary material for: Implementation of Telemental Health Services Before COVID-19: Rapid Umbrella Review of Systematic Reviews
Source: J Med Internet Res. 2021 Jul 20;23(7):e26492. doi: 10.2196/26492 (PMC8335619; doi:10.2196/26492)
Supplement: Multimedia Appendix 1 [file jmir_v23i7e26492_app1.docx]

# Appendix 1: Search strategy

| **Psychinfo** | **604** |
| --- | --- |
| **CENTRAL systematic reviews** | **94** |
| **Pub med** | **543** |
| **Total** | **1241** |
| **Dupes removed** | **1086** |

**All searches 1^st^ January 2010-August 26^th^ 2020**

**PsychINFO**

[*reviews]*

1. (systematic or structured or evidence or trials or studies).ti. and ((review or overview or look or examination or update* or summary).ti. or review.pt.)
2. meta-analysis.pt. or (meta-analys* or meta analys* or metaanalys* or meta synth* or meta-synth* or metasynth*).ti,ab,id,hw.
3. ((systematic or meta) adj2 (analys* or review)).ti,id. or ((systematic* or quantitativ* or methodologic* or qualitative) adj2 (review* or overview*)).ti,ab,id,sh. or ((quantitativ* or qualitativ*) adj2 synthesis*).ti,ab,id,hw.
4. (integrative research review* or research integration).ti,ab. or (review.ti,id,pt. and (trials as topic or studies as topic).hw.) or (evidence adj3 review*).ti,ab,id. or (realist adj3 review*).ti,ab,id.
5. (OR/1-5)

[*mental disorders]*

1. mental disorders/ or anxiety disorders/ or obsessive compulsive disorder/ or panic disorder/ or phobias/ or social phobia/ or bipolar disorder/ or eating disorders/ or anorexia nervosa/ or binge eating disorder/ or bulimia/ or affective disorders/ or major depression/ or (exp personality disorders/) or schizophrenia/ or affective psychosis/ or catatonic schizophrenia / or paranoid schizophrenia/ or body dysmorphic disorder/ or posttraumatic stress disorder/ or delusions/ or dysthymic disorder/ or endogenous depression/ or reactive depression/ or recurrent depression/ or treatment resistant depression/ or atypical depression/ or “depression (emotion)”/ or self-injurious behavior/ or suicidal ideation/ or attempted suicide/
2. alzheimer's disease/ or exp dementia/
3. (affective disorder* or agoraphobi* or anorexia nervosa or anxiety or BPD or binge eat* or binging or bipolar or bulimi* or combat disorder* or compulsi* or delusion* or depersonali#ation or depressed or depression or depressive or eating disorder* or EDNOS or emotional trauma or mania or manic or mood? or neurotic or obsess* or panic or paranoi* or parasuicid* or personality disorder* or phobi* or ((post-trauma* or posttrauma*) adj stress*) or psychiatr* or psychopathol* or psychosomatic or psychotic or psychos* or PTSD or schizo* or (self adj (injur* or harm or mutilat*)) or social anxiety or suicid*).ti,id,hw.
4. Mental health/ or ((mental* or psychiatric) adj2 (health* or ill* or disorder* or diagnos?s or problem*)) .ti,ab,id.
5. Mental health services/ or community mental health services/ or community psychiatry/ or (mental health service? or CAMHS or "child and adolescent mental health service?" or psychiatry or psychology or psychotherap*).ti,ab,id.
6. (OR/6-10)

[*remote working]*

1. telecommunications/ or telepsychiatry/ or telemedicine/ or computer assisted therapy/ or telephone/ or technology/ or videoconferencing/ or internet/ or computer mediated communication/ or computers/ or exp online therapy/
2. *Answering service*.ti, id.*
3. ((mobile* or phone? or telephone? or remote* or distan* or online or virtual or electronic or email or e-mail or video) adj3 (consult* or counsel* or follow up or follow-up or support* or interview* or monitor* or therap* or treatment? or "CBT")).ti,ab,id.
4. (ehealth* or e-health* or emedicine* or e-medicine* or etherap* or e-therap* or mhealth or m-health or m health or eCBT or e-CBT or iCBT or i-CBT or Interap* or (electronic adj2 CBT) or telemedicine or telecare or telepsychiatry or telecommunications or teleconferencing or computer assisted therap* or teletherap* or telemental or tele-mental).ti,ab,id.
5. *(videoconferen* or video-conferen* or videophone? Or video-phone? or video-call* or video-based call* or video call* or video based call*).ti, ab, id.*
6. *Digital interventions/*
7. *Exp electronic communication/*
8. *Mobile devices/ or mobile health/ or mobile technology/*
9. *Teleconferencing/*
10. *(OR/12-20)*
11. *5 AND 11 AND 21*

*Restrict: 2010-2020*

**PUB MED**

**("systematic review"[Title/Abstract] OR "literature review"[Title/Abstract] OR "narrative review"[Title] OR "qualitative review"[Title] OR "evidence review"[Title] OR "systematic quantitative review"[Title] OR "meta review"[Title] OR "systematic critical review"[Title] OR "realist review"[Title] OR"systematic cochrane review"[Title] OR "systematic search and review"[Title] OR "systematic integrative review"[Title] or "qualitative synthesis"[Title] or "narrative synthesis"[Title] or meta-synthesis*[Title/Abstract]) AND (("Dementia"[MeSH Terms] OR "Alzheimer Disease"[MeSH Terms]) OR ("Mental Health"[Title/Abstract] OR "mental problem*"[Title/Abstract] OR "mental disorder*"[Title/Abstract] OR "mental illness*"[Title/Abstract] OR "Depression"[Title/Abstract] OR "depressive disorder*"[Title/Abstract] OR "Anxiety"[Title/Abstract] OR "anxiety disorder*"[Title/Abstract] OR "phobi*"[Title/Abstract] OR "agoraphobi*"[Title/Abstract] OR "anxious"[Title/Abstract] OR "obsess*"[Title/Abstract] OR "compulsi*"[Title/Abstract] OR "panic"[Title/Abstract] OR "PTSD"[Title/Abstract] OR "post traumatic stress"[Title/Abstract] OR "posttraumatic stress"[Title/Abstract] OR "stress disorder*"[Title/Abstract] OR "psychiatr*"[Title/Abstract] OR "SMI"[Title/Abstract] OR "psycho*"[Title/Abstract] OR "schizo*"[Title/Abstract] OR "manic"[Title/Abstract] OR "mania"[Title/Abstract] OR "bipolar"[Title/Abstract] OR "personality disorder*"[Title/Abstract] OR "self-harm"[Title/Abstract] OR "self-injury"[Title/Abstract] OR "self-harm"[Title/Abstract] OR "self-injury"[Title/Abstract] OR "psychological disorder"[Title/Abstract] OR "Psychiatric illness"[Title/Abstract] OR "psychiatric disorder*"[Title/Abstract]) OR ("Anxiety Disorders"[MeSH Terms] OR "Bipolar Disorder"[MeSH Terms] OR "Feeding and Eating Disorders"[MeSH Terms] OR "Depressive Disorder"[MeSH Terms] OR "Neurotic Disorders"[MeSH Terms] OR "Personality Disorders"[MeSH Terms] OR "Psychotic Disorders"[MeSH Terms] OR "Schizophrenia"[MeSH Terms] OR "Mental Disorders"[MeSH Terms:noexp] OR "Mental Health"[MeSH Terms] OR "Mentally Ill Persons"[MeSH Terms] OR "self-injurious behavior"[MeSH Terms] OR "psychology, clinical"[MeSH Terms]) OR ("Mental Health Services"[MeSH Terms]) OR ("mental health service*"[Title/Abstract] OR "CAMHS"[Title/Abstract] OR "psychiatry"[Title/Abstract] OR "psychology"[Title/Abstract] OR "psychotherap*"[Title/Abstract])) AND (("Telemedicine"[MeSH Terms:noexp] OR "Remote Consultation"[MeSH Terms] OR "Distance Counseling"[MeSH Terms] OR "therapy, computer-assisted"[MeSH Terms] OR "Videoconferencing"[MeSH Terms] OR "internet-based intervention"[MeSH Terms]) OR ("ehealth*"[Title/Abstract] OR "e health*"[Title/Abstract] OR "emedicine*"[Title/Abstract] OR "e medicine*"[Title/Abstract] OR "etherap*"[Title/Abstract] OR "e therap*"[Title/Abstract] OR "eCBT"[Title/Abstract] OR "e-CBT"[Title/Abstract] OR "iCBT"[Title/Abstract] OR "i-CBT"[Title/Abstract] OR "interap*"[all fields] OR "telemedicine"[Title/Abstract] OR "telecare"[Title/Abstract] OR "telepsychiatry"[Title/Abstract] OR "telecommunication*"[Title/Abstract] OR "teleconferencing"[Title/Abstract] OR "computer assisted therap*"[Title/Abstract] OR "teletherap*"[Title/Abstract] OR "telemental"[Title/Abstract] OR "e-mental health"[Title/Abstract] OR "e mental health"[Title/Abstract] OR "videoconferen*"[Title/Abstract] OR "video conferen*"[Title/Abstract]** OR “video-call*”[Title/Abstract] OR “Video call*”[Title/Abstract] or video-based call*[Title/Abstract] OR “video based call*”[Title/Abstract] **OR "videophone*"[Title/Abstract] OR "video phone*"[Title/Abstract]) OR (("mobile*"[Title/Abstract] OR "phone*"[Title/Abstract] OR "telephone*"[Title/Abstract] OR "remote*"[Title/Abstract] OR "distan*"[Title/Abstract] OR "online"[Title/Abstract] OR "virtual"[Title/Abstract] OR "electronic"[Title/Abstract] OR "email"[Title/Abstract] OR "e-mail"[Title/Abstract]) N2 ("consult*"[Title/Abstract] OR "counsel*"[Title/Abstract] OR "support*"[Title/Abstract] OR "interview*"[Title/Abstract] OR "monitor*"[Title/Abstract] OR "therap*"[Title/Abstract] OR "treatment*"[Title/Abstract] OR "CBT"[Title/Abstract])))**

*Limit to 2010 onwards.***Cochrane Systematic review database**

| 1 | MeSH descriptor: [Feeding and Eating Disorders] this term only |
| --- | --- |
| 2 | MeSH descriptor: [Anorexia Nervosa] this term only |
| 3 | MeSH descriptor: [Bulimia Nervosa] this term only |
| 4 | MeSH descriptor: [Binge-Eating Disorder] this term only |
| 5 | MeSH descriptor: [Bulimia] this term only |
| 6 | ("eating disorder*" or ` (eat* near/3 mood*) or EDNOS or anorexi* or orthorexi* or bulimi* or diabulimi* or (bing* near/2 (eat* or purg*))):ti,ab,kw |
| 7 | MeSH descriptor: [Mood Disorders] this term only |
| 8 | MeSH descriptor: [Depressive Disorder] this term only |
| 9 | MeSH descriptor: [Depressive Disorder, Major] this term only |
| 10 | MeSH descriptor: [Seasonal Affective Disorder] this term only |
| 11 | MeSH descriptor: [Dysthymic Disorder] this term only |
| 12 | MeSH descriptor: [Depression] this term only |
| 13 | (mood* or depress* or dysthymi* or "affective disorder*" or "affective symptom*"):ti,ab,kw |
| 14 | MeSH descriptor: [Anxiety Disorders] explode all trees |
| 15 | (general* near/2 anxi*):ti,ab,kw |
| 16 | anxiety:ti |
| 17 | ("anxiety disorder*" or "social* anxiety" or phobi* or agoraphobi* or anxious or obsess* or compulsi* or panic or PTSD or "post traumatic stress" or "posttraumatic stress" or neurosis or neuroses or neurotic):ti,ab,kw |
| 18 | ((psychological or emotional) near/2 (debrief* or stress* or trauma*)):ti,kw |
| 19 | MeSH descriptor: [Obsessive Behavior] this term only |
| 20 | MeSH descriptor: [Self-Injurious Behavior] explode all trees |
| 21 | ((self next (injur* or mutilat*)) or suicide* or suicidal or parasuicid* or para-suicid*):ti,ab,kw |
| 22 | MeSH descriptor: [Somatoform Disorders] explode all trees |
| 23 | ((conduct or behavi* or antisocial or anti-social or dyssocial or emotional* or internalizing or internalising or externalizing or externalising) near/2 (disorder* or problem* or difficult* or disturb* or psychopath*)):ti,ab,kw |
| 24 | MeSH descriptor: [Personality Disorders] explode all trees |
| 25 | (BPD or personality disorder*):ti,ab,kw |
| 26 | #1 OR #2 OR #3 OR #4 OR #5 OR #6 OR #7 OR #8 OR #9 OR #10 OR #11 OR #12 OR #13 OR #14 or #15 or #16 OR #17 OR #18 OR #19 OR #20 OR #21 OR #22 OR #23 OR #24 OR #25 |
| 27 | MeSH descriptor: [Dementia] explode all trees |
| 28 | MeSH descriptor: [Alzheimer Disease] 2 tree(s) exploded |
| 29 | MeSH descriptor: [Mental Health Services] explode all trees |
| 30 | MeSH descriptor: [Psychiatry] explode all trees |
| 31 | MeSH descriptor: [Psychotherapy] explode all trees |
| 32 | ("mental health service*" or CAMHS or "child and adolescent mental health service*" or psychiatry or psychology or psychotherap*):ti,ab,kw |
| 33 | #27 OR #28 OR #29 OR #30 OR #31 OR #32 |
| 34 | MeSH descriptor: [Telecommunications] explode all trees |
| 35 | MeSH descriptor: [Technology] this term only |
| 36 | MeSH descriptor: [Answering Services] explode all trees |
| 37 | MeSH descriptor: [Distance Counseling] explode all trees |
| 38 | MeSH descriptor: [Internet-Based Intervention] explode all trees |
| 39 | ((mobile* or phone* or telephone* or remote* or distan* or online or virtual or electronic or email or e-mail or video) N3 (consult* or counsel* or follow up or follow-up or support* or interview* or monitor* or therap* or treatment? or "CBT")):ti,ab,kw |
| 40 | (ehealth* or e-health* or emedicine* or e-medicine* or etherap* or e-therap* or eCBT or e-CBT or iCBT or i-CBT or Interap* or mhealth or m health or m-health or (electronic N2 CBT) or telemedicine or telecare or telepsychiatry or telecommunications or teleconferencing or computer assisted therap* or teletherap* or telemental or tele mental or tele-mental):ti,ab,kw |
| 41 | (videoconferen* or video-conferen* or videophone* Or video-phone* or video call* or video-call* or video call* or (video N2 call*) or (video-based N2 call*)):ti,ab,kw |
| 42 | #34 OR #35 OR #36 OR #37 OR #38 OR #39 OR #40 OR #41 |
| 43 | (#26 OR #33) AND #42 [limit to cochrane review database, 2010-2020] |
