# Supplementary material for: Implementation of Telemental Health Services Before COVID-19: Rapid Umbrella Review of Systematic Reviews
Source: J Med Internet Res. 2021 Jul 20;23(7):e26492. doi: 10.2196/26492 (PMC8335619; doi:10.2196/26492)
Supplement: Multimedia Appendix 2 [file jmir_v23i7e26492_app2.docx]

# Appendix 2: Study Overlap

| Primary Study^a^ | Berryhill 2019a | Berryhill 2019b | Bolton 2015 | Christensen 2019 | Coughtrey 2018 | Dorstyn 2013 | Drago 2016 | Garcia-Lizana 2010 | Harerimana 2019 | Hassan 2019 | Lin 2019 | Lins 2014 | Muskens 2014 | Naslund 2020 | Norwood 2018 | Olthius 2016a | Othius 2016b | Turgoose 2018 | N reviews included |
| --- | --- | --- | --- | --- | --- | --- | --- | --- | --- | --- | --- | --- | --- | --- | --- | --- | --- | --- | --- |
| Aburizik 2013 |  |  |  |  |  |  |  |  | X |  |  |  |  |  |  |  |  |  | 1 |
| Acierno 2016 |  |  |  |  |  |  |  |  |  |  |  |  |  |  |  |  | X | X | 2 |
| Acierno 2017 |  |  |  |  |  |  |  |  |  |  |  |  |  |  |  |  |  | X | 1 |
| Ahmed 2008 |  |  |  |  |  |  |  |  |  |  |  |  |  | X |  |  |  |  | 1 |
| Amarendran 2011 |  |  |  |  |  |  | X |  |  |  |  |  |  |  |  |  |  |  | 1 |
| Andersson 2005 |  |  |  |  |  |  |  |  |  |  |  |  | X |  |  |  |  |  | 1 |
| Andersson 2009 |  |  |  |  |  |  |  |  |  |  |  |  |  |  |  | X |  |  | 1 |
| Andersson 2012 |  |  |  |  |  |  |  |  |  |  |  |  |  |  |  | X |  |  | 1 |
| Andersson 2012 |  |  |  |  |  |  |  |  |  |  |  |  |  |  |  | X |  |  | 1 |
| Andersson 2013 |  |  |  |  |  |  |  |  |  |  |  |  |  |  |  | X |  |  | 1 |
| Arnaert 2007 |  |  |  | X |  |  |  |  |  |  |  |  |  |  |  |  |  |  | 1 |
| Aziz 2004 |  |  |  |  |  |  |  |  |  |  |  |  | X |  |  |  |  |  | 1 |
| Baca 2007 |  |  |  |  |  |  |  |  |  |  | X |  |  |  |  |  |  |  | 1 |
| Barerra-Valencia 2017 |  |  |  |  |  |  |  |  |  |  |  |  |  | X |  |  |  |  | 1 |
| Berger 2009 |  |  |  |  |  |  |  |  |  |  |  |  |  |  |  | X |  |  | 1 |
| Berger 2011 |  |  |  |  |  |  |  |  |  |  |  |  |  |  |  | X |  |  | 1 |
| Berger 2014 |  |  |  |  |  |  |  |  |  |  |  |  |  |  |  | X |  |  | 1 |
| Bergstrom 2010 |  |  |  |  |  |  |  |  |  |  |  |  |  |  |  | X |  |  | 1 |
| Bishop 2002 |  |  |  | X |  |  |  | X |  | X |  |  |  |  |  |  |  |  | 3 |
| Bouchard 2004 |  | X |  |  |  |  |  | X |  | X |  |  |  |  | X |  |  |  | 4 |
| Brøndbo 2012 |  |  |  |  |  |  | X |  |  |  |  |  |  |  |  |  |  |  | 1 |
| Brooks 2013 |  |  |  |  |  |  |  |  |  |  |  |  |  |  |  |  |  | X | 1 |
| Burke 1995 |  |  |  |  |  |  |  |  |  |  |  |  | X |  |  |  |  |  | 1 |
| Butler 2012 |  |  |  |  |  |  |  |  |  |  |  |  |  | X |  |  |  |  | 1 |
| Cacciola 1999 |  |  |  |  |  |  |  |  |  |  |  |  | X |  |  |  |  |  | 1 |
| Carlbring 2004 |  |  |  |  |  |  |  |  |  |  |  |  |  |  |  | X |  |  | 1 |
| Carlbring 2006 |  |  |  |  |  |  |  |  |  |  |  |  |  |  |  | X |  |  | 1 |
| Carlbring 2007 |  |  |  |  |  |  |  |  |  |  |  |  |  |  |  | X |  |  | 1 |
| Carlson 2012 |  |  |  |  |  |  |  |  |  |  | X |  |  |  |  |  |  |  | 1 |
| Cernvall 2015 |  |  |  |  |  |  |  |  |  |  |  |  |  |  |  |  | X |  | 1 |
| Chang 1999 |  |  |  |  |  |  |  |  |  |  |  | X |  |  |  |  |  |  | 1 |
| Chang 2004 |  |  |  |  |  |  |  |  |  |  |  | X |  |  |  |  |  |  | 1 |
| Chang 2018 |  |  |  |  |  |  |  |  |  |  | X |  |  |  |  |  |  |  | 1 |
| Chiu 2009 |  |  |  |  |  | X |  |  |  |  |  |  |  |  |  |  |  |  | 1 |
| Choi 2012 |  |  |  |  |  | X |  |  |  |  |  |  |  |  |  |  |  |  | 1 |
| Choi 2014 |  |  |  |  |  |  |  | X |  |  |  |  |  |  |  |  |  |  | 1 |
| Choi 2014 |  |  |  |  |  |  | X |  | X |  |  |  |  |  |  |  |  |  | 2 |
| Choi 2014 |  |  |  | X |  |  | X |  |  |  |  |  |  |  |  |  |  |  | 2 |
| Chong 2012 |  |  |  |  |  | X |  |  |  | X |  |  |  |  |  |  |  |  | 2 |
| Clapp 2016 |  |  |  |  |  |  |  |  |  |  |  |  |  |  |  |  |  | X | 1 |
| Conn 2013 |  |  |  | X |  |  |  |  | X |  |  |  |  |  |  |  |  |  | 2 |
| Cowain 2001 |  | X |  |  |  |  |  |  |  |  |  |  |  |  |  |  |  |  | 1 |
| Crippa 2008 |  |  |  |  |  |  |  |  |  |  |  |  | X |  |  |  |  |  | 1 |
| Crowe 2016 |  |  |  | X |  |  |  |  |  |  |  |  |  |  |  |  |  |  | 1 |
| Davis 2004 |  |  |  |  |  |  |  |  |  |  |  | X |  |  |  |  |  |  | 1 |
| De Las Cuevas 2003 |  |  |  | X |  |  |  |  |  |  |  |  |  |  |  |  |  |  | 1 |
| De Las Cuevas 2006 |  |  |  |  |  |  | X | X |  | X |  |  |  |  |  |  |  |  | 3 |
| De Leo 2014 |  |  |  |  |  |  |  |  |  |  | X |  |  |  |  |  |  |  | 1 |
| Demiris 2011 |  | X |  |  |  |  |  |  |  |  |  |  |  |  |  |  |  |  | 1 |
| Dobkin 2011 |  |  |  |  | x |  |  |  |  |  |  |  |  |  |  |  |  |  | 1 |
| DuHamel 2010 |  |  |  |  |  |  |  |  |  |  |  |  |  |  |  |  | X |  | 1 |
| Dunstan 2012 |  | X |  |  |  |  |  |  |  |  |  |  |  |  |  |  |  |  | 1 |
| Dwight-Johnson 2011 |  |  |  |  | x | X |  |  |  |  |  |  |  |  |  |  |  |  | 2 |
| Egede 2016 |  |  |  | X |  |  |  |  |  |  |  |  |  |  |  |  |  |  | 1 |
| Egede 2017 |  |  |  |  |  |  |  |  |  |  |  |  |  | X |  |  |  |  | 1 |
| Eibl 2017 |  |  |  |  |  |  |  |  |  |  | X |  |  |  |  |  |  |  | 1 |
| Eisdorfer 2003 |  |  |  |  |  | X |  |  |  |  |  |  |  |  |  |  |  |  | 1 |
| Elford 2000 |  |  |  |  |  |  | X |  |  | X |  |  |  |  |  |  |  |  | 2 |
| Elford 2001 |  |  |  |  |  |  |  |  |  |  |  |  |  | X |  |  |  |  | 1 |
| Engel 2015 |  |  |  |  |  |  |  |  |  |  |  |  |  |  |  |  | X |  | 1 |
| Evans 2004 |  |  |  |  |  |  |  |  |  |  |  |  | X |  |  |  |  |  | 1 |
| Finkel 2007 |  |  |  |  |  |  |  |  |  |  |  | X |  |  |  |  |  |  | 1 |
| Fitt 2012 |  | X |  |  |  |  |  |  |  |  |  |  |  |  |  |  |  |  | 1 |
| Fortney 2007 |  |  |  | X |  |  | X |  |  |  |  |  |  |  |  |  |  |  | 2 |
| Fortney 2013 |  |  |  |  |  |  | X |  |  | X |  |  |  |  |  |  |  |  | 2 |
| Fortney 2015 |  |  |  |  |  |  |  |  |  |  |  |  |  |  |  |  |  | X | 1 |
| Frank 2017 |  |  |  | X |  |  |  |  |  |  |  |  |  |  |  |  |  |  | 1 |
| Franklin 2016 |  |  |  |  |  |  |  |  |  |  |  |  |  |  |  |  | X | X | 2 |
| Frueh 2007 |  |  |  |  |  |  |  | X |  | X |  |  |  |  |  |  | X | X | 4 |
| Frueh 2005 |  |  |  |  |  |  |  |  |  |  | X |  |  |  |  |  |  |  | 1 |
| Frueh 2007 |  |  |  |  |  |  |  | X |  |  |  |  |  |  |  |  |  |  | 1 |
| Furmark 2009 |  |  |  |  |  |  |  |  |  |  |  |  |  |  |  | X |  |  | 1 |
| Gant 2007 |  |  |  |  |  |  |  |  |  |  |  | X |  |  |  |  |  |  | 1 |
| Garzon-maldonado 2017 |  |  |  |  |  |  |  |  |  |  |  |  |  | X |  |  |  |  | 1 |
| Gerlach-Reinholz 2017 |  |  |  |  |  |  |  |  |  |  |  |  |  | X |  |  |  |  | 1 |
| Germain 2009 |  | X | X |  |  |  |  |  |  |  |  |  |  |  | X |  |  |  | 3 |
| Glueckauf 2012 |  |  |  |  |  | X |  |  |  |  |  | X |  |  |  |  |  |  | 2 |
| Godelski 2012 |  |  |  |  |  |  |  |  | X |  |  |  |  |  |  |  |  |  | 1 |
| Gonzalez 2015 |  |  |  |  |  |  |  |  |  |  |  |  |  |  |  |  |  |  | 0 |
| Greene 2010 |  |  |  |  |  |  |  |  |  |  |  |  |  |  |  |  |  | X | 1 |
| Greenwood 2004 |  |  |  | X |  |  |  |  |  |  |  |  |  |  |  |  |  |  | 1 |
| Griffiths 2006 |  | X |  |  |  |  |  |  |  |  |  |  |  |  |  |  |  |  | 1 |
| Gros 2011 |  | X | X |  |  |  |  |  |  |  |  |  |  |  |  |  |  | X | 3 |
| Gros 2012 |  |  |  |  |  |  |  |  |  |  |  |  |  |  |  |  |  |  | 0 |
| Gros 2016 |  |  |  |  |  |  |  |  |  |  |  |  |  |  |  |  |  | X | 1 |
| Grubbs 2015 |  |  |  |  |  |  |  |  |  |  |  |  |  |  |  |  |  | X | 1 |
| Grubbs 2017 |  |  |  |  |  |  |  |  |  |  |  |  |  |  |  |  |  | X | 1 |
| Hajebi 2012 |  |  |  |  |  |  |  |  |  |  |  |  | X |  |  |  |  |  | 1 |
| Hassija 2011 |  |  |  |  |  |  |  |  |  |  |  |  |  |  |  |  |  |  | 0 |
| Hedman 2011 |  |  |  |  |  |  |  |  |  |  |  |  |  |  |  | X |  |  | 1 |
| Hernandez-Tejada 2014 |  |  |  |  |  |  |  |  |  |  |  |  |  |  |  |  |  | X | 1 |
| Hilty 2007 |  |  |  | X |  |  |  |  |  | X |  |  |  |  |  |  |  |  | 2 |
| Himelhoch 2011 |  |  |  |  | x |  |  |  |  |  |  |  |  |  |  |  |  |  | 1 |
| Himle 2006 |  |  |  |  |  |  |  |  |  |  |  |  |  |  | X |  |  |  | 1 |
| Hull 2017 |  |  |  |  |  |  |  |  |  |  |  |  |  | X |  |  |  |  | 1 |
| Ivarsson 2014 |  |  |  |  |  |  |  |  |  |  |  |  |  |  |  | X | X |  | 2 |
| Jaconis 2017 |  |  |  |  |  |  |  |  |  |  |  |  |  |  |  |  |  | X | 1 |
| Jang 2014 |  |  |  | X |  | X |  |  |  |  |  |  |  |  |  |  |  |  | 2 |
| Johnston 2011 |  |  |  |  |  |  |  |  |  |  |  |  |  |  |  | X |  |  | 1 |
| Jones 2012 |  |  |  |  |  |  |  |  |  |  |  |  |  | X |  |  |  |  | 1 |
| Jones 2014 |  |  |  |  |  |  |  |  |  |  |  |  |  | X |  |  |  |  | 1 |
| Jong 2004 |  |  |  |  |  |  |  |  |  |  |  |  |  | X |  |  |  |  | 1 |
| Kennedy 2000 |  |  |  |  |  |  |  |  |  |  |  |  |  | X |  |  |  |  | 1 |
| Kim 2016 |  |  |  |  |  |  |  |  |  |  | X |  |  |  |  |  |  |  | 1 |
| King 2009 |  |  |  |  |  |  |  |  |  |  | X |  |  |  |  |  |  |  | 1 |
| King 2014 |  |  |  |  |  |  |  |  |  |  | X |  |  |  |  |  |  |  | 1 |
| Kiropoulos 2008 |  |  |  |  |  |  |  |  |  |  |  |  |  |  |  | X |  |  | 1 |
| Klee 2016 |  |  |  |  |  |  |  |  |  |  |  |  |  |  |  |  |  | X | 1 |
| Klein 2010 |  |  | X |  |  |  |  |  |  |  |  |  |  |  |  |  |  |  | 1 |
| Knaevelsrud 2015 |  |  |  |  |  |  |  |  |  |  |  |  |  |  |  |  | X |  | 1 |
| Kobak 2008 |  |  |  | X |  |  |  |  |  |  |  |  |  |  |  |  |  |  | 1 |
| Kobak 2015 |  |  |  |  |  |  |  |  |  |  |  |  |  |  |  | X |  |  | 1 |
| Kok 2014 |  |  |  |  |  |  |  |  |  |  |  |  |  |  |  | X |  |  | 1 |
| Lazzari 2011 |  |  |  |  |  |  |  |  |  |  |  |  |  |  |  |  |  |  | 0 |
| Lewis 2013 |  |  | X |  |  |  |  |  |  |  |  |  |  |  |  |  |  |  | 1 |
| Lichstein 2013 |  |  |  |  |  |  |  |  | X |  |  |  |  |  | X |  |  |  | 2 |
| Lightstone 2015 |  |  |  |  |  |  |  |  |  |  |  |  |  |  |  |  |  | X | 1 |
| Lindsay 2015 |  |  |  |  |  |  |  |  |  |  |  |  |  |  |  |  |  | X | 1 |
| Littleton 2012 |  |  | X |  |  |  |  |  |  |  |  |  |  |  |  |  |  |  | 1 |
| Litz 2007 |  |  | X |  |  |  |  |  |  |  |  |  |  |  |  |  | X |  | 2 |
| Lovell 2000 |  |  |  |  | X |  |  |  |  |  |  |  |  |  |  |  |  |  | 1 |
| Luxton 2015 |  | X |  |  |  |  |  |  |  |  |  |  |  |  |  |  |  | X | 2 |
| Luxton 2016 |  | X |  | X |  |  |  |  |  |  |  |  |  |  |  |  |  |  | 2 |
| Lyneham 2005 |  |  |  |  |  |  |  |  |  |  |  |  | X |  |  |  |  |  | 1 |
| Maieritsch 2015 |  |  |  |  |  |  | X |  |  |  |  |  |  |  |  |  | X | X | 3 |
| Malhotra 2014 |  |  |  |  |  |  | X |  |  |  |  |  |  |  |  |  |  |  | 1 |
| Manchanda 1998 |  |  |  |  |  |  |  |  |  |  |  |  |  |  | X |  |  |  | 1 |
| Manguno-Mire 2007 |  |  |  |  |  |  |  | X |  | X |  |  |  |  |  |  |  |  | 2 |
| Marchand 2011 |  | X | X |  |  |  |  |  |  |  |  |  |  |  |  |  |  |  | 2 |
| Matsuura 2000 |  |  |  |  |  |  | X |  |  |  |  |  |  |  |  |  |  |  | 1 |
| Mclellan 2017 |  | X |  |  |  |  |  |  |  |  |  |  |  |  |  |  |  |  | 1 |
| Menon 2001 |  |  |  | X |  |  |  |  |  |  |  |  |  |  |  |  |  |  | 1 |
| Miller 2002 |  |  |  |  | x |  |  |  |  |  |  |  |  |  |  |  |  |  | 1 |
| Miller 2016 |  |  |  |  |  |  |  |  |  |  |  |  |  |  |  |  |  | X | 1 |
| Mitchell 2008 |  |  |  |  |  |  | X | X |  | X |  |  |  |  | X |  |  |  | 4 |
| Modai 2006 |  |  |  |  |  |  |  |  |  |  |  |  |  | X |  |  |  |  | 1 |
| Mohr 2000 |  |  |  |  | x |  |  |  |  |  |  |  |  |  |  |  |  |  | 1 |
| Mohr 2005 |  |  |  |  | x |  |  |  |  |  |  |  |  |  |  |  |  |  | 1 |
| Mohr 2006 |  |  |  |  | x |  |  |  |  |  |  |  |  |  |  |  |  |  | 1 |
| Mohr 2011 |  |  |  |  | x |  |  |  |  |  |  |  |  |  |  |  |  |  | 1 |
| Mohr 2013 |  |  |  |  |  |  |  |  | X |  |  |  |  |  |  |  |  |  | 1 |
| Moreno 2012 |  |  |  |  |  | X | X |  |  |  |  |  |  |  |  |  |  |  | 2 |
| Morland 2004 |  |  |  |  |  |  |  |  |  |  |  |  |  |  |  |  |  | X | 1 |
| Morland 2010 |  |  |  |  |  |  | X |  |  |  |  |  |  |  |  |  |  | X | 2 |
| Morland 2011 |  |  |  |  |  |  |  |  |  |  |  |  |  |  |  |  |  | X | 1 |
| Morland 2013 |  |  | X |  |  |  |  |  |  |  |  |  |  | X |  |  |  |  | 2 |
| Morland 2014 |  |  |  |  |  |  | X |  |  |  |  |  |  |  |  |  | X | X | 3 |
| Morland 2015 |  |  |  |  |  |  |  |  |  |  |  |  |  |  |  |  |  | X | 1 |
| Morland 2015 |  |  |  |  |  |  |  |  |  |  |  |  |  |  |  |  |  | X | 1 |
| Morland 2015 |  |  |  |  |  |  |  |  |  |  |  |  |  |  | X |  | X | X | 3 |
| Munro Cullum 2014 |  |  |  |  |  |  | X |  |  |  |  |  |  |  |  |  |  |  | 1 |
| Nelson 2003 |  |  |  |  |  |  |  | X |  | X |  |  |  |  |  |  |  |  | 2 |
| Neufeld 2013 |  |  |  |  |  |  |  |  |  |  |  |  |  | X |  |  |  |  | 1 |
| Newby 2013 |  |  |  |  |  |  |  |  |  |  |  |  |  |  |  | X |  |  | 1 |
| Nieminen 2016 |  |  |  |  |  |  |  |  |  |  |  |  |  |  |  |  | X |  | 1 |
| Niles 2012 |  |  |  |  |  |  |  |  |  |  |  |  |  |  |  |  | X | X | 2 |
| Nordgren 2014 |  |  |  |  |  |  |  |  |  |  |  |  |  |  |  | X |  |  | 1 |
| O’Reilly 2007 |  |  |  | X |  |  |  | X |  | X |  |  |  |  |  |  |  |  | 3 |
| Ojserkis 2013 |  | X |  |  |  |  |  |  |  |  |  |  |  |  |  |  |  |  | 1 |
| Olthuis 2015 |  |  |  |  | X |  |  |  |  |  |  |  |  |  |  |  |  |  | 1 |
| Paing 2010 |  |  |  |  |  |  |  |  |  |  |  |  | X |  |  |  |  |  | 1 |
| Painter 2017 |  |  |  |  |  |  |  |  |  |  |  |  |  | X |  |  |  |  | 1 |
| Paulsen 1988 |  |  |  |  |  |  |  |  |  |  |  |  | X |  |  |  |  |  | 1 |
| Paxling 2011 |  |  |  |  |  |  |  |  |  |  |  |  |  |  |  | X |  |  | 1 |
| Poon 2005 |  |  |  |  |  |  | X |  |  |  |  |  |  |  |  |  |  |  | 1 |
| Price |  |  |  |  |  |  |  |  |  |  |  |  |  |  |  |  |  | X | 1 |
| Pyne 2010 |  |  |  |  |  |  |  |  |  |  |  |  |  | X |  |  |  |  | 1 |
| Rabinowitz 2010 |  |  |  |  |  |  |  |  |  |  |  |  |  | X |  |  |  |  | 1 |
| Ransom 2008 |  |  |  |  | x |  |  |  |  |  |  |  |  |  |  |  |  |  | 1 |
| Revicki 1997 |  |  |  |  |  |  |  |  |  |  |  |  | X |  |  |  |  |  | 1 |
| Richter 2015 |  |  |  |  |  |  |  |  |  |  | X |  |  |  |  |  |  |  | 1 |
| Robinson 2010 |  |  |  |  |  |  |  |  |  |  |  |  |  |  |  | X |  |  | 1 |
| Rohde 1997 |  |  |  |  |  |  |  |  |  |  |  |  | X |  |  |  |  |  | 1 |
| Ruskin 2004 |  |  |  |  |  |  | X | X |  | X |  |  |  | X |  |  |  |  | 4 |
| Russell 2015 |  |  |  |  |  |  |  |  | X |  |  |  |  |  |  |  |  |  | 1 |
| Salfi 2004 |  |  |  |  |  |  |  |  |  |  |  | X |  |  |  |  |  |  | 1 |
| Schutte 2015 |  |  |  |  |  |  | X |  |  |  |  |  |  |  |  |  |  |  | 1 |
| Seidel 2014 |  |  |  |  |  |  | X |  |  |  |  |  |  |  |  |  |  |  | 1 |
| Shealy 2015 |  | X |  |  |  |  |  |  |  |  |  |  |  |  |  |  |  |  | 1 |
| Shore 2007 |  |  |  |  |  |  | X |  |  |  |  |  |  |  |  |  |  |  | 1 |
| Shore 2012 |  |  |  |  |  |  |  |  |  |  |  |  |  |  |  |  |  | X | 1 |
| Shore 2014 |  |  |  | X |  |  |  |  |  |  |  |  |  |  |  |  |  |  | 1 |
| Simon 1993 |  |  |  |  |  |  |  |  |  |  |  |  | X |  |  |  |  |  | 1 |
| Simon 2009 |  |  |  |  |  |  |  |  |  |  |  |  |  | X |  |  |  |  | 1 |
| Simpson 2001 |  |  |  |  |  |  |  |  |  |  |  |  |  | X |  |  |  |  | 1 |
| Simpson 2001 |  |  |  | X |  |  |  |  |  |  |  |  |  |  |  |  |  |  | 1 |
| Simpson 2006 |  |  |  |  |  |  |  |  |  |  |  |  |  |  |  |  |  |  | 0 |
| Singh 2007 |  |  |  |  |  |  | X |  |  |  |  |  |  |  |  |  |  |  | 1 |
| Smith 2007 |  |  |  |  |  |  |  |  |  |  |  |  |  | X |  |  |  |  | 1 |
| Smolenski 2017 |  |  |  |  |  |  |  |  |  |  |  |  |  |  |  |  |  |  | 0 |
| Spaniel 2015 |  |  |  |  |  |  |  |  |  | X |  |  |  |  |  |  |  |  | 1 |
| Spaulding 2010 |  |  |  |  |  |  |  |  |  |  |  |  |  | X |  |  |  |  | 1 |
| Spence 2011 |  |  | X |  |  |  |  |  |  |  |  |  |  |  |  |  | X |  | 2 |
| Spence 2013 |  |  | X |  |  |  |  |  |  |  |  |  |  |  |  |  |  |  | 1 |
| Spence 2014 |  |  |  |  |  |  |  |  |  |  |  |  |  |  |  |  | X |  | 1 |
| Staton-Tindall 2014 |  |  |  |  |  |  |  |  |  |  | X |  |  |  |  |  |  |  | 1 |
| Stefan 2013 |  |  |  |  |  |  |  |  |  |  |  |  |  |  | X |  |  |  | 1 |
| Steffen 2000 |  |  |  |  |  |  |  |  |  |  |  | X |  |  |  |  |  |  | 1 |
| Stevens 1999 |  |  |  |  |  |  |  | X |  |  |  |  |  |  |  |  |  |  | 1 |
| Strachan 2012 |  | X |  |  |  |  |  |  |  |  |  |  |  |  |  |  | X | X | 3 |
| Stubbings 2013 |  | X |  |  |  |  |  |  |  |  |  |  |  |  | X |  |  |  | 2 |
| Swinson 1995 |  |  |  |  | X |  |  |  |  |  |  |  |  |  |  |  |  |  | 1 |
| Tan 2013 |  |  |  |  |  |  |  |  |  |  |  |  |  |  |  |  |  | X | 1 |
| Tang 2001 |  |  |  | X |  |  |  |  | X |  |  |  |  | X |  |  |  |  | 3 |
| Tarp 2017 |  |  |  |  |  |  |  |  |  |  | X |  |  |  |  |  |  |  | 1 |
| Taylor 2003 |  |  |  |  | X |  |  |  |  |  |  |  |  |  |  |  |  |  | 1 |
| Théberge-Lapointe 2015 |  | X |  |  |  |  |  |  |  |  |  |  |  |  |  |  |  |  | 1 |
| Thorp 2012 |  |  |  |  |  |  |  |  |  |  |  |  |  |  |  |  |  | X | 1 |
| Titov 2008 |  |  |  |  |  |  |  |  |  |  |  |  |  |  |  | X |  |  | 1 |
| Titov 2008 |  |  |  |  |  |  |  |  |  |  |  |  |  |  |  | X |  |  | 1 |
| Titov 2008 |  |  |  |  |  |  |  |  |  |  |  |  |  |  |  | X |  |  | 1 |
| Titov 2009 |  |  |  |  |  |  |  |  |  |  |  |  |  |  |  | X |  |  | 1 |
| Titov 2010 |  |  |  |  |  |  |  |  |  |  |  |  |  |  |  | X |  |  | 1 |
| Titov 2011 |  |  |  |  |  |  |  |  |  |  |  |  |  |  |  | X |  |  | 1 |
| Tremont 2008 |  |  |  |  |  |  |  |  |  |  |  | X |  |  |  |  |  |  | 1 |
| Tse 2015 |  |  |  |  |  |  | X |  |  |  |  |  |  |  |  |  |  |  | 1 |
| Tuerk 2010 |  |  | X |  |  |  |  |  |  |  |  |  |  |  |  |  |  | X | 2 |
| Tunstall 1997 |  |  |  |  |  |  |  |  |  |  |  |  | X |  |  |  |  |  | 1 |
| Tutty 2010 |  |  |  |  | x |  |  |  |  |  |  |  |  |  |  |  |  |  | 1 |
| Uebelacker 2011 |  |  |  |  |  | X |  |  |  |  |  |  |  |  |  |  |  |  | 1 |
| Vahia 2015 |  |  |  |  |  |  | X |  |  |  |  |  |  |  |  |  |  |  | 1 |
| Van Ballegooijen 2013 |  |  |  |  |  |  |  |  |  |  |  |  |  |  |  | X |  |  | 1 |
| van Bastelaar 2011 |  |  |  |  |  |  |  |  | X |  |  |  |  |  |  |  |  |  | 1 |
| Ward-King 2010 |  |  |  |  |  |  |  |  |  |  |  |  | X |  |  |  |  |  | 1 |
| Watson 1992 |  |  |  |  |  |  |  |  |  |  |  |  | X |  |  |  |  |  | 1 |
| Wells 1988 |  |  |  |  |  |  |  |  |  |  |  |  | X |  |  |  |  |  | 1 |
| Whealin 2015 |  |  |  |  |  |  |  |  |  |  |  |  |  |  |  |  |  | X | 1 |
| Wierwille 2016 |  |  |  |  |  |  |  |  |  |  |  |  |  |  |  |  |  | X | 1 |
| Wilz 2011 |  |  |  |  |  |  |  |  |  |  |  | X |  |  |  |  |  |  | 1 |
| Wims 2010 |  |  |  |  |  |  |  |  |  |  |  |  |  |  |  | X |  |  | 1 |
| Winter 2007 |  |  |  |  |  |  |  |  |  |  |  | X |  |  |  |  |  |  | 1 |
| Wray 2010 |  |  |  |  |  |  |  |  |  |  |  |  |  | X |  |  |  |  | 1 |
| Yeung 2009 |  |  |  | X |  |  |  |  |  |  |  |  |  |  |  |  |  |  | 1 |
| Yoshino 2001 |  |  |  |  |  |  | X |  |  |  |  |  |  |  |  |  |  |  | 1 |
| Yuen 2013 |  | X |  |  |  |  |  |  |  |  |  |  |  |  | X |  |  |  | 2 |
| Yuen 2015 |  | X |  |  |  |  |  |  |  |  |  |  |  |  |  |  | X | X | 3 |
| Zheng 2014 |  |  |  |  |  |  |  |  |  |  |  |  |  |  |  |  |  |  | 0 |
| Zheng 2017 |  |  |  |  |  |  |  |  |  |  | X |  |  |  |  |  |  |  | 1 |
| Ziemba 2014 |  | X | X |  |  |  |  |  |  |  |  |  |  |  |  |  | X | X | 4 |
| Note. Sansom Daley 2016's included papers are not listed here. The review included guidance only and so there was no study overlap.  ^a^ Refer to appendix 2b for full study reference | | | | | | | | | | | | | | | | | | |  |
