# Supplementary material for: Implementation of Telemental Health Services Before COVID-19: Rapid Umbrella Review of Systematic Reviews
Source: J Med Internet Res. 2021 Jul 20;23(7):e26492. doi: 10.2196/26492 (PMC8335619; doi:10.2196/26492)
Supplement: Multimedia Appendix 3 [file jmir_v23i7e26492_app3.docx]

# Appendix 3: Primary studies full title

Aburizik 2013. A pilot randomized controlled trial of a depression and disease management programme delivered by phone.

Acierno 2016. Behavioural activation and therapeutic exposure for posttraumatic stress disorder: A noninferiority trial of treatment delivered in person versus home-based telehealth.

Acierno 2017. A non-inferiority trial of Prolonged Exposure for posttraumatic stress disorder: In person versus home-based telehealth.

Ahmed 2008. Feasibility of epilepsy follow-up care through telemedicine: a pilot study on the patient's perspective.

Amarendran 2011. The reliability of telepsychiatry for a neuropsychiatric assessment.

Andersson 2005. Internet-based self-help for depression: Randomised controlled trial

Andersson 2009. Internet-based self-help versus one-session exposure in the treatment of spider phobia: A randomized controlled trial.

Andersson 2012. Internet-based psychodynamic versus cognitive behavioural guided self-help for generalized anxiety disorder: A randomized controlled trial.

Andersson 2012. Therapist experience and knowledge acquisition in internet-delivered CBT for social anxiety disorder: a randomized controlled trial.

Andersson 2013. Internet-based exposure treatment versus one-session exposure treatment of snake phobia: A randomized controlled trial.

Arnaert 2007. Attitudes towards videotelephones: An exploratory study of older adults with depression.

Aziz 2004. Comparability of telephone and face-to-face interviews in assessing patients with posttraumatic stress disorder.

Baca 2007. Satisfaction with long-distance motivational interviewing for problem drinking.

Barerra-Valencia 2017. Cost-effectiveness of synchronous vs. asynchronous telepsychiatry in prison inmates with depression.

Berger 2009. Internet-based treatment for social phobia: A randomized controlled trial.

Berger 2011. Internet-based treatment of social phobia: A randomized controlled trial comparing unguided with two types of guided self-help.

Berger 2014. Internet-based guided self-help for several anxiety disorders: A randomized controlled trial comparing a tailored with a standardized disorder-specific approach.

Bergstrom 2010. Internet-versus group-administered cognitive behaviour therapy for panic disorder in a psychiatric setting: A randomised trial.

Bishop 2002. Client satisfaction in a feasibility study comparing face-to-face interviews with telepsychiatry.

Bouchard 2004. Delivering cognitive-behaviour therapy for panic disorder with agoraphobia in videoconference.

Brøndbo 2012. Agreement on web-based diagnoses and severity of mental health problems in Norwegian child and adolescent mental health services.

Brooks 2013. Reaching rural communities with culturally appropriate care: A model for adapting remote monitoring to American Indian veterans with posttraumatic stress disorder.

Burke 1995. The reliability and validity of the Geriatric Depression Rating Scale administered by telephone.

Butler 2012. Cost analysis of store-and-forward telepsychiatry as a consultation model for primary care.

Cacciola 1999. Comparability of telephone and in-person structured clinical interview for DSM-III-R (SCID) diagnoses.

Carlbring 2004. Treatment of panic disorder: Live therapy vs. self-help via the internet.

Carlbring 2006. Remote treatment of panic disorder: A randomized trial of internet-based cognitive behaviour therapy supplemented with telephone calls.

Carlbring 2007. Treatment of social phobia: Randomised trial of internet-delivered cognitive-behavioural therapy with telephone support.

Carlson 2012. Telehealth-delivered group smoking cessation for rural and urban participants: Feasibility and cessation rates.

Cernvall 2015. Internet-based guided self-help for parents of children on cancer treatment: A randomized controlled trial.

Chang 1999. Cognitive-behavioural intervention for homebound caregivers of persons with dementia.

Chang 2004. Perceived helpfulness of telephone calls.

Chang 2018. Expanding access to buprenorphine treatment in rural areas with telemedicine.

Chiu 2009. Internet-based care-giver support for Chinese Canadians taking care of a family member with Alzheimer disease and related dementia.

Choi 2012. culturally attuned Internet treatment for depression amongst Chinese Australians: a randomised controlled trial.

Choi 2014. Acceptance of home-based telehealth problem-solving therapy for depressed low income homebound older adults: qualitative interviews with the participants and aging-service case managers.

Choi 2014. Six month postintervention depression and disability outcomes of in-home telehealth problem-solving therapy for depressed, low-income homebound older adult.

Choi 2014. Telehealth problem-solving therapy for depressed low-income homebound older adults.

Chong 2012. Feasibility and acceptability of clinic-based telepsychiatry for low-income Hispanic primary care patients.

Clapp 2016. Patterns of change in response to prolonged exposure: Implications for treatment outcome.

Conn 2013. Program evaluation of a telepsychiatry service for older adults connecting a university-affiliated geriatric centre to a rural psychogeriatric outreach service in Northwest Ontario.

Cowain 2001. Cognitive-behavioural therapy via videoconferencing to a rural area.

Crippa 2008. Comparability between telephone and face-to-face structured clinical interview for DSM-IV in assessing social anxiety disorder.

Crowe 2016. A pilot program in rural telepsychiatry for deaf and hard of hearing populations.

Davis 2004. A comparison of in-home and telephone-based skill training interventions with caregivers of persons with dementia.

De Las Cuevas 2003. Telepsychiatry in the Canary Islands: User acceptance and satisfaction.

De Las Cuevas 2006. Randomized clinical trial of telepsychiatry through videoconference versus face-to-face conventional psychiatric treatment.

De Leo 2014. A brief behavioural telehealth intervention for veterans with alcohol misuse problems in VA primary care.

Demiris 2011. Use of videophones to deliver a cognitive-behavioural therapy to hospice caregivers.

Dobkin 2011. Telephone-based cognitive-behavioural therapy for depression in Parkinson disease.

DuHamel 2010. Randomized clinical trial of telephone-administered cognitive-behavioural therapy to reduce post-traumatic stress disorder and distress symptoms after hematopoietic stem-cell transplantation.

Dunstan 2012. Treatment via videoconferencing: a pilot study of delivery by clinical psychology trainees.

Dwight-Johnson 2011. Telephone-based cognitive-behavioural therapy for Latino patients living in rural areas: a randomized pilot study.

Egede 2016. Psychotherapy for depression in older veterans via telemedicine: Effect on quality of life, satisfaction, treatment credibility, and service delivery perception.

Egede 2017. Trajectory of cost overtime after psychotherapy for depression in older veterans via telemedicine.

Eibl 2017. The effectiveness of telemedicine-delivered opioid agonist therapy in a supervised clinical setting.

Eisdorfer 2003. The effect of a family therapy and technology-based intervention on caregiving depression.

Elford 2000. A randomized, controlled trial of child psychiatric assessments conducted using videoconferencing.

Elford 2001. A prospective satisfaction study and cost analysis of a pilot child telepsychiatry service in Newfoundland.

Engel 2015. Delivery of self-training and education for stressful situations(DESTRESS-PC): A randomized trial of nurse assisted online self-management for PTSD in primary care.

Evans 2004. Assessing mental health in primary care research using standardized scales: can it be carried out over the telephone?

Finkel 2007. E-care: a telecommunications technology intervention for family caregivers of dementia patients.

Fitt 2012. Metacognitive therapy for obsessive compulsive disorder by videoconference: a preliminary study.

Fortney 2007. A randomized trial of telemedicine-based collaborative care for depression.

Fortney 2013. Practice-based versus telemedicine-based collaborative care for depression in rural federally qualified health centres: a pragmatic randomized comparative effectiveness trial.

Fortney 2015. Telemedicine based collaborative care for posttraumatic stress disorder: a randomized clinical trial

Frank 2017. Video conference-based psychotherapeutic follow-up treatment. qualitative case study using CBASP approach.

Franklin 2016. Face to face but not in the same place: A pilot study of prolonged exposure therapy.

Frueh 2007. A randomized trial of telepsychiatry for post-traumatic stress disorder.

Frueh 2005. Telehealth service delivery for persons with alcoholism.

Frueh 2007. Therapist adherence and competence with manualized cognitive-behavioural therapy for PTSD delivered via videoconferencing technology.

Furmark 2009. Guided and unguided self-help for social anxiety disorder: Randomised controlled trial.

Gant 2007. Comparative outcomes of two distance-based interventions for male caregivers of family members with dementia.

Garzon-maldonado 2017. An assessment of telephone assistance systems for caregivers of patients with Alzheimer’s disease.

Gerlach-Reinholz 2017. Telefoncoaching bei depression [telephone coaching for depression].

Germain 2009. Effectiveness of cognitive behavioural therapy administered by videoconference for post-traumatic stress disorder.

Glueckauf 2012. Telephone-based, cognitive-behavioural therapy for African American dementia caregivers with depression: initial findings.

Godelski 2012. Home telemental health implementation and outcomes using electronic messaging.

Gonzalez 2015. Telehealth videoconferencing psychotherapy in rural primary care.

Greene 2010. How does tele-mental health affect group therapy process? Secondary analysis of a noninferiority trial.

Greenwood 2004. Evaluation of a rural telepsychiatry service.

Griffiths 2006. Telemedicine as a means of delivering cognitive-behavioural therapy to rural and remote mental health clients.

Gros 2011. Exposure therapy for PTSD delivered to veterans via telehealth: predictors of treatment completion and outcome and comparison to treatment delivered in person.

Gros 2012. Behavioural activation and therapeutic exposure: An investigation of relative symptom changes in PTSD and depression during the course of integrated behavioural activation, situational exposure, and imaginal exposure techniques.

Gros 2016. Treatment satisfaction of home-based telehealth versus in person delivery of prolonged exposure for combat-related PTSD in veterans.

Grubbs 2015. Predictors of initiation and engagement of cognitive processing therapy among veterans with PTSD enrolled in collaborative care.

Grubbs 2017. Usual care for rural veterans with posttraumatic stress disorder.

Hajebi 2012. Telephone versus face-to-face administration of the structured clinical interview for diagnostic and statistical manual of mental disorders, fourth edition, for diagnosis of psychotic disorders.

Hassija 2011. The effectiveness and feasibility of videoconferencing technology to provide evidence-based treatment to rural domestic violence and sexual assault populations.

Hedman 2011. Internet-based cognitive behaviour therapy vs. cognitive behavioural group therapy for social anxiety disorder: A randomized controlled non-inferiority trial.

Hernandez-Tejada 2014. Early treatment withdrawal from evidence-based psychotherapy for PTSD: Telemedicine and in-person parameters.

Hilty 2007. A randomized controlled trial of disease management modules, including telepsychiatric care for depression in rural primary care.

Himelhoch 2011. Feasibility of telephone- based cognitive behavioural therapy targeting major depression among urban dwelling African-American people with co-occurring HIV.

Himle 2006. Videoconferencing-based cognitive-behavioural therapy for obsessive-compulsive disorder.

Hull 2017. A study of asynchronous mobile-enabled SMS text psychotherapy.

Ivarsson 2014. Guided internet-delivered cognitive behaviour therapy for post-traumatic stress disorder: A randomized controlled trial.

Jaconis 2017. Concurrent treatment of PTSD and alcohol use disorder via telehealth in a female Iraq veteran.

Jang 2014. Telecounselling for the linguistically isolated: a pilot study with older Korean immigrants

Johnston 2011. A RCT of a transdiagnostic internet-delivered treatment for three anxiety disorders: Examination of support roles and disorder specific outcomes.

Jones 2012. Acceptability and cost-effectiveness of military telehealth mental health screening.

Jones 2014. Technology-enhanced program for child disruptive behaviour disorders: development and pilot randomized control trial.

Jong 2004. Managing suicides via videoconferencing in a remote northern community in Canada.

Kennedy 2000. A community-based approach to evaluation of health outcomes and costs for telepsychiatry in a rural population.

Kim 2016. A randomized controlled trial of a videoconferencing smoking cessation intervention for Korean American women: Preliminary findings.

King 2009. Assessing the effectiveness of an Internet-based videoconferencing platform for delivering intensified substance abuse counselling.

King 2014. A randomized trial of web-based videoconferencing for substance abuse counselling.

Kiropoulos 2008. Is internet-based CBT for panic disorder and agoraphobia as eGective as face-to-face CBT?

Klee 2016. Interest in technology based therapies hampered by access: A survey of veterans with serious mental illnesses.

Klein 2010. A therapist-assisted cognitive behaviour therapy for posttraumatic stress disorder: pre-, post-and 3-month follow-up results from an open trial.

Knaevelsrud 2015. Web-based psychotherapy for posttraumatic stress disorder in war-traumatized Arab patients: Randomized controlled trial.

Kobak 2008. Face-to-face versus remote administration of the Montgomery-Asberg Depression Rating Scale using videoconference and telephone.

Kobak 2015. Computer-assisted cognitive behaviour therapy for obsessive compulsive disorder: A randomized trial on the impact of lay vs. professional coaching.

Kok 2014. Short term effectiveness of web-based guided self-help for phobic outpatients: Randomized controlled trial.

Lazzari 2011. Behavioural activation treatment for depression in older adults delivered via videoconferencing: A pilot study.

Lewis 2013. Development of a guided self-help (GSH) program for the treatment of mild to-moderate Posttraumatic Stress Disorder (PTSD).

Lichstein 2013. Telehealth cognitive behaviour therapy for co-occurring insomnia and depression symptoms in older adults in the united states.

Lightstone 2015. Collaborative music therapy via remote video technology to reduce a veteran’s symptoms of severe, chronic PTSD.

Lindsay 2015. Implementation of video telehealth to improve access to evidence-based psychotherapy for posttraumatic stress disorder.

Littleton 2012. From survivor to thriver: a pilot study of an online program for rape victims.

Litz 2007. A randomized, controlled proof of concept trial of an internet-based therapist assisted self-management treatment for posttraumatic stress disorder.

Lovell 2000. Telephone treatment of obsessive-compulsive disorder.

Luxton 2015. An evaluation of the feasibility and safety of a home-based telemental health treatment for post-traumatic stress in the U.S. Military.

Luxton 2016. Home-based tele behavioural health for US military personnel and veterans with depression: A RCT.

Lyneham 2005. Agreement between telephone and in-person delivery of a structured interview for anxiety disorders in children.

Maieritsch 2015. Randomized controlled equivalence trial comparing videoconference and in person delivery of cognitive processing therapy for PTSD.

Malhotra 2014. Development of a novel diagnostic system for a tele psychiatric application: a pilot validation study.

Manchanda 1998. Cognitive behaviour therapy via interactive video.

Manguno-Mire 2007. The use of telemedicine to evaluate competency to stand trial: a preliminary randomized controlled study.

Marchand 2011. Relative efficacy of cognitive-behavioural therapy administered by videoconference for posttraumatic stress disorder: A six-month follow-up.

Matsuura 2000. Application of telepsychiatry: a preliminary study.

Mclellan 2017. Delivery of a therapist-facilitated telecare anxiety program to children in rural communities: a pilot study.

Menon 2001. Evaluation of a portable low-cost videophone system in the assessment of depressive symptoms and cognitive function in elderly mentally ill veterans.

Miller 2002. Interpersonal psychotherapy delivered over the telephone to recurrent depressives: A pilot study.

Miller 2016. Interest in use of technology for healthcare among veterans receiving treatment for mental health.

Mitchell 2008. A randomized trial comparing the efficacy of cognitive-behavioural therapy for bulimia nervosa delivered via telemedicine versus face-to-face.

Modai 2006. Cost-effectiveness, safety, and satisfaction with video telepsychiatry versus face-to-face care in ambulatory settings.

Mohr 2000. Telephone administered cognitive-behavioural therapy for the treatment of depressive symptoms in multiple sclerosis.

Mohr 2005. Telephone-administered psychotherapy for depression.

Mohr 2006. Telephone administered cognitive behavioural therapy for the treatment of depression in a rural primary care clinic.

Mohr 2011. Telephone-administered cognitive behavioural therapy for veterans served by community-based outpatient clients.

Mohr 2013. A randomized controlled trial evaluating a manualized TeleCoaching protocol for improving compliance with a web-based intervention for the treatment of depression.

Moreno 2012. Use of standard webcam and Internet equipment for tele-psychiatry treatment of depression among underserved Hispanics.

Morland 2004. Telemedicine and coping skills groups for Pacific Island veterans with 584 Journal of Telemedicine and Telecare.

Morland 2010. Telemedicine for anger management therapy in a rural population of combat veterans with posttraumatic stress disorder: a randomized noninferiority trial.

Morland 2011. Group cognitive processing therapy delivered to veterans via telehealth: A pilot cohort.

Morland 2013. Telemedicine: a cost-reducing means of delivering psychotherapy to rural combat veterans with PTSD.

Morland 2014. Cognitive processing therapy for posttraumatic stress disorder delivered to rural veterans via telemental health: a randomized noninferiority clinical trial.

Morland 2015. Telemedicine versus in-person delivery of cognitive processing therapy for women with posttraumatic stress disorder: A randomized noninferiority trial.

Morland 2015. Telemedicine versus in‐person delivery of cognitive processing therapy for women with posttraumatic stress disorder: A randomized noninferiority trial.

Morland 2015. Telemedicine versus in-person delivery of cognitive processing therapy for women with posttraumatic stress disorder: A randomized noninferiority trial.

Munro Cullum 2014. Teleneuropsychology: evidence for video teleconference-based neuropsychological assessment.

Nelson 2003. Treating childhood depression over videoconferencing.

Neufeld 2013. Walk-in telemental health clinics improve access and efficiency: a 2-year follow-up analysis.

Newby 2013. Internet cognitive behavioural therapy for mixed anxiety and depression: A randomized controlled trial and evidence of effectiveness in primary care.

Nieminen 2016. Internet-provided cognitive behaviour therapy of posttraumatic stress symptoms following childbirth – A randomized controlled trial.

Niles 2012. Comparing mindfulness and psychoeducation treatments for combat-related PTSD using a telehealth approach.

Nordgren 2014. Effectiveness and cost-effectiveness of individually tailored Internet-delivered cognitive behaviour therapy for anxiety disorders in a primary care population: A randomized controlled trial.

O’Reilly 2007. Is telepsychiatry equivalent to face-to-face psychiatry? Results from a randomized controlled equivalence trial.

Ojserkis 2013. Paediatric obsessive-compulsive disorder.

Olthuis 2015. Telephone delivered cognitive behavioural therapy for high anxiety sensitivity: A randomised controlled trial.

Paing 2010. Face-to-face versus telephone administration of the parent’s version of the children’s interview for psychiatric syndromes (P-ChIPS).

Painter 2017. Cost-effectiveness of telemedicine-based collaborative care for post-traumatic stress disorder.

Paulsen 1988. Reliability of the telephone interview in diagnosing anxiety disorders.

Paxling 2011. Guided internet-delivered cognitive behaviour therapy for generalized anxiety disorder: A randomized controlled trial.

Poon 2005. Cognitive intervention for community dwelling older persons with memory problems: telemedicine versus face-toface treatment.

Price. Examination of prior experience with telehealth and comfort with telehealth technology as a moderator of treatment response for PTSD and depression in veterans.

Pyne 2010. Cost-effectiveness analysis of a rural telemedicine collaborative care intervention for depression.

Rabinowitz 2010. Benefits of a telepsychiatry consultation service for rural nursing home residents.

Ransom 2008. Telephone delivered, interpersonal psychotherapy for HIV-infected rural persons with depression: A pilot trial.

Revicki 1997. Telephone versus in-person clinical and health status assessment interviews in patients with bipolar disorder.

Richter 2015. Comparative and cost effectiveness of telemedicine versus telephone counselling for smoking cessation.

Robinson 2010. Internet treatment for generalized anxiety disorder: A randomized controlled trial comparing clinician vs. technician assistance.

Rohde 1997. Comparability of telephone and face-to-face interviews in assessing axis I and II disorders.

Ruskin 2004. Treatment outcomes in depression: comparison of remote treatment through telepsychiatry to in-person treatment.

Russell 2015. Exploring the predictors of home telehealth uptake by elderly Australian healthcare consumers.

Salfi 2004. Seeking to understand telephone support for dementia caregivers: a qualitative case study.

Schutte 2015. Usability and reliability of a remotely administered adult autism assessment, the autism diagnostic observation schedule (ADOS) module 4.

Seidel 2014. Agreement between telepsychiatry assessment and face-to-face assessment for Emergency Department psychiatry patients.

Shealy 2015. Delivering an evidence-based mental health treatment to underserved populations using telemedicine: the case of a trauma-affected adolescent in a rural setting.

Shore 2007. Diagnostic reliability of telepsychiatry in American Indian veterans.

Shore 2012. Characteristics of telemental health service use by American Indian veterans.

Shore 2014. Meeting veterans where they're @: a VA-based Telemental Health (HBTMH) pilot program.

Simon 1993. Telephone assessment of depression severity.

Simon 2009. Incremental benefit and cost of telephone care management and telephone psychotherapy doe depression in primary care.

Simpson 2001. Evaluation of a routine telepsychiatry service.

Simpson 2001. Telepsychiatry as a routine service-the perspective of the patient.

Simpson 2006. Does video therapy work? A single case series of bulimic disorders.

Singh 2007. Accuracy of telepsychiatric assessment of new routine outpatient referrals.

Smith 2007. A cost-minimization analysis of a telepaediatric mental health service for patients in rural and remote Queensland.

Smolenski 2017. Unobserved heterogeneity in response to treatment for depression through videoconference.

Spaniel 2015. Psychiatrist's adherence: a new factor in relapse prevention in schizophrenia A randomized controlled study on relapse control through telemedicine system.

Spaulding 2010. Cost savings of telemedicine utilization for child psychiatry in a rural Kansas community.

Spence 2011. Randomized controlled trial of internet-delivered cognitive behavioural therapy for Posttraumatic Stress Disorder.

Spence 2013. Internet-delivered eye movement desensitization and reprocessing: and open trial

Spence 2014. Internet-based trauma-focused cognitive behavioural therapy for PTSD with and without exposure components: A randomized controlled trial.

Staton-Tindall 2014. METelemedicine: A pilot study with rural alcohol users on community supervision.

Stefan 2013. Face‐to‐face counselling versus high definition holographic projection system. Efficacy and therapeutic alliance. A brief research report.

Steffen 2000. Anger management for dementia caregivers: a preliminary study using video and telephone interventions.

Stevens 1999. Pilot study of televideo psychiatric assessments in an underserviced community.

Strachan 2012. An integrated approach to delivering exposure-based treatment for symptoms of PTSD and depression in OIF/OEF veterans: preliminary findings.

Stubbings 2013. Comparing in-person to videoconference-based cognitive behavioural therapy for mood and anxiety disorders: randomized controlled trial.

Swinson 1995. Efficacy of telephone- administered behavioural therapy for panic disorder with agoraphobia.

Tan 2013. Improving access to care for women veterans suffering from chronic pain and depression associated with trauma.

Tang 2001. Telepsychiatry in psychogeriatric service: a pilot study.

Tarp 2017. Effectiveness of optional videoconferencing-based treatment of alcohol use disorders: Randomized controlled trial.

Taylor 2003. Telephone-administered cognitive-behaviour therapy for obsessive-compulsive disorder.

Théberge-Lapointe 2015. Efficacy of a cognitive-behavioural therapy administered by videoconference for generalized anxiety disorder.

Thorp 2012. Lessons learned from studies of psychotherapy for posttraumatic stress disorder via video teleconferencing.

Titov 2008. Shyness 1: Distance treatment of social phobia over the internet.

Titov 2008. Shyness 2: Treating social phobia online: Replication and extension.

Titov 2008. Shyness 3: Randomized controlled trial of guided versus unguided internet-based CBT for social phobia.

Titov 2009. Clinician-assisted internet based treatment is effective for generalized anxiety disorder: Randomized controlled trial.

Titov 2010. Transdiagnostic internet treatment for anxiety disorders: A randomized controlled trial.

Titov 2011. Transdiagnostic internet treatment for anxiety and depression: A randomised controlled trial.

Tremont 2008. Telephone delivered psychosocial intervention reduces burden in dementia caregivers.

Tse 2015. Teletherapy delivery of caregiver behaviour training for children with attention-deficit hyperactivity disorder.

Tuerk 2010. A pilot study of prolonged exposure therapy for Posttraumatic Stress Disorder delivered via telehealth technology.

Tunstall 1997. Concurrent validity of a telephone-administered version of the Gospel Oak instrument (including the SHORT-CARE).

Tutty 2010. Evaluating the effectiveness of cognitive-behavioural teletherapy in depressed adults.

Uebelacker 2011. Telephone depression care management for Latino Medicaid health plan members: a pilot randomized controlled trial.

Vahia 2015. Telepsychiatry for neurocognitive testing in older rural Latino adults.

Van Ballegooijen 2013. An Internet-based guided self-help intervention for panic symptoms: Randomized controlled trial.

van Bastelaar 2011. Web-based depression treatment for type 1 and type 2 diabetic patients: a randomized, controlled trial.

Ward-King 2010. Brief report: telephone administration of the autism diagnostic interview– revised: reliability and suitability for use in research.

Watson 1992. Comparability of telephone and face to face diagnostic interview schedules.

Wells 1988. Agreement between face-to-face and telephone-administered versions of the depression section of the NIMH diagnostic interview schedule.

Whealin 2015. E-mental health preferences of veterans with and without probable posttraumatic stress disorder.

Wierwille 2016. Effectiveness of PTSD telehealth treatment in a VA clinical sample.

Wilz 2011. Goal attainment and treatment compliance in a cognitive-behavioural telephone intervention for family caregivers of persons with dementia.

Wims 2010. Clinician-assisted internet-based treatment is effective for panic: A randomized controlled trial.

Winter 2007. Evaluation of a telephone-based support group intervention for female caregivers of community dwelling individuals with dementia.

Wray 2010. The effect of telephone support groups on costs of care for veterans with dementia.

Yeung 2009. Feasibility and effectiveness of telepsychiatry services for Chinese immigrants in a nursing home.

Yoshino 2001. Telepsychiatry: assessment of televideo psychiatric interview reliability with present- and next-generation Internet infrastructures.

Yuen 2013. Acceptance based behaviour therapy for social anxiety disorder through videoconferencing.

Yuen 2015. Randomized controlled trial of home-based telehealth versus in-person prolonged exposure for combat-related PTSD in veterans: preliminary results.

Zheng 2014. Telehealth-based therapy connecting rural Mandarin-speaking traumatized clients with a Mandarin-speaking therapist.

Zheng 2017. Treatment outcome comparison between telepsychiatry and face-to-face buprenorphine medication-assisted treatment for opioid use disorder: A 2-year retrospective data analysis.

Ziemba 2014. Posttraumatic stress disorder treatment for operation enduring freedom/operation Iraqi freedom com-bat veterans through a civilian community-based telemedicine network.
