# Supplementary material for: Implementation of Telemental Health Services Before COVID-19: Rapid Umbrella Review of Systematic Reviews
Source: J Med Internet Res. 2021 Jul 20;23(7):e26492. doi: 10.2196/26492 (PMC8335619; doi:10.2196/26492)
Supplement: Multimedia Appendix 4 [file jmir_v23i7e26492_app4.docx]

# Appendix 4: Quality assessment further detail

| AMSTAR2 Criteria | Harerimana 2019 | Dorstyn 2013 | Berryhill 2019a | Berryhill 2019b | Bolton 2015 | Christensen 2019 | Coughtrey 2018 | Drago 2016 | Garcia-Lizana 2010 | Hassan 2019 | Lin  2019 | Lins  2014 | Muskens 2014 | Norwood 2018 | Olthuis 2016a | Olthuis 2016b | Sansom-Daly 2016 | Turgoose 2018 |
| --- | --- | --- | --- | --- | --- | --- | --- | --- | --- | --- | --- | --- | --- | --- | --- | --- | --- | --- |
| 1 | Yes | Yes | Yes | Yes | Yes | yes | Yes | Yes | Yes | Yes | Yes | Yes | Yes | Yes | Yes | Yes | No | No |
| 2 | Yes | No | No | No | No | No | No | No | No | No | No | Partial yes | No | No | Yes | No | No | No |
| 3 | Yes | No | No | No | Yes | Yes | Yes | Yes | No | Yes | Yes | Yes | Yes | Yes | Yes | Yes | Yes | Yes |
| 4 | No | Partial yes | No | No | Yes | Partial yes | Yes | Yes | Partial yes | Yes | Yes | Yes | Yes | Yes | Partial yes | Partial yes | Yes | No |
| 5 | No | No | No | No | No | No | No | No | Yes | No | No | Yes | Yes | No | Yes | No | Yes | No |
| 6 | No | No | Yes | Yes | No | Yes | Yes | Yes | Yes | Yes | Yes | Partial yes | Yes | No | Yes | Yes | No | No |
| 7 | No | No | No | No | No | No | No | No | No | No | No | Yes | No | No | Yes | No | No | No |
| 8 | Yes | Partial yes | Yes | Yes | Yes | Partial yes | Partial yes | Yes | Partial yes | Partial yes | Yes | Partial yes | Partial yes | Partial yes | Yes | Partial yes | Yes | Yes |
| 9 | Yes | No | Yes | Yes | No | Partial yes | Yes | Partial yes | No | No | Yes | Yes | Yes | yes | Yes | Yes | Yes | No |
| 10 | No | No | No | No | No | No | Yes | Yes | No | No | No | Yes | No | No | No | No | No | No |
| 11 | No MA | No MA | No MA | No MA | Yes | No MA | No MA | Yes | No MA | No MA | No MA | Yes | No MA | Yes | Yes | Yes | No MA | No MA |
| 12 | No MA | No MA | No MA | No MA | Partial yes | No MA | No MA | No | No MA | No MA | No MA | Yes | No MA | Yes | No | No | No MA | No MA |
| 13 | No | No | No | No | No | No | No | No | No | No | Yes | Yes | Yes | yes | Yes | Yes | No | No |
| 14 | Yes | No | No | No | No | No | Yes | Yes | No | No | Yes | Yes | Yes | Yes | Yes | Yes | Yes | Yes |
| 15 | No MA | No MA | No MA | No MA | Yes | No MA | No MA | Yes | No MA | No MA | No MA | No | No MA | No | Yes | Yes | No MA | No MA |
| 16 | Yes | No | Yes | Yes | Yes | Yes | Yes | Yes | Yes | Yes | Yes | Yes | Yes | Yes | Yes | No | Yes | Yes |
| *MA: Meta-Analysis* | | | | | | | | | | | | | | | | | | |
| *1. PICO criteria included*  *2. Explicit statement that the review methods were established prior to the conduct of the review*  *3 Selection of study designs to include explained*  *4. Comprehensive literature search strategy*  *5.Study selection performed in duplicate*  *6. Data extraction performed in duplicate*  *7. List of excluded studies and reasons provided*  *8. Included studies described in adequate detail*  *9.Satisfactory assessment of risk of bias (RoB) in individual studies*  *10. Sources of funding reported*  *11. Meta-Analysis: appropriate methods for statistical combination of results*  *12. Meta-Analysis: Assessment of the potential impact of RoB in individual studies*  *13. Interpreation accounts for RoB*  *14. Satisfactory explanation for, and discussion of, any heterogeneity observed in the results of the review*  *15. Meta-Analysis: Adequate investigation of publication bias*  *16. Potential conflicts of interest reported* | | | | | | | | | | | | | | | | | | |
