# Supplementary material for: Implementation of Telemental Health Services Before COVID-19: Rapid Umbrella Review of Systematic Reviews
Source: J Med Internet Res. 2021 Jul 20;23(7):e26492. doi: 10.2196/26492 (PMC8335619; doi:10.2196/26492)
Supplement: Multimedia Appendix 5 [file jmir_v23i7e26492_app5.docx]

# Appendix 5: Guideline recommendations (Sansom-Daley 2016)

|  | Guidelines |
| --- | --- |
| *Appropriateness of e-mental health* | Client related factors: Firm recommendations that mental health professionals should incorporate an assessment process to determine the appropriateness of e-mental health services for an individual client (58%, 11/19 guidelines). However, only four sets of guidelines provided more concrete recommendations as to how professionals could undertake such an assessment Service related factors: Tentative recommendations that psychological tests designed to be implemented face to face may not be possible or ethical to conduct online. |
| *Competence* | Competence:  1) Firm recommendations that mental health professionals should provide online services within the boundaries of their competence, with an understanding of the limits and applications of different technologies. (58%, 11/19 guidelines).  2) Firm recommendations that professionals should acquire skills to manage technology they are using (53%, 10/19 guidelines).  3) Tentative recommendations that mental health professionals should be culturally competent to deliver online services to different populations, including considerations of clients' ethnic/racial, cultural, linguistic, gender/sexual orientation, geographic, and socioeconomic backgrounds. (42%, 8/19 guidelines). |
| *Legal and regulatory issues* | 1) Firm recommendations that professionals should know and comply with all relevant laws and regulations (79%, 15/19 guidelines) 2) Firm recommendations that professionals should ensure that their licensing board approves of the provision of online services, and obtain site-specific credentialing across jurisdictions where necessary (63%, 12/19)  3) Tentative recommendations that professionals delivering e-mental health interventions should adhere to the usual laws and professional standards applicable to record keeping, particularly where the intervention diverges from usual practice (47%, 9/19 guidelines).  4) Tentative recommendations that professionals should take steps to determine the age of potential clients to establish the appropriateness of e-mental health interventions, and should ensure that a parent/guardian’s consent is obtained for all minors before services proceed (21%, 4/19 guidelines) |
| *Confidentiality* | 1) Firm recommendation that mental health professionals should take all up to date precautionary efforts to protect clients' confidentiality using e-mental health services (63%, 12/19 guidelines).  2) Tentative recommendations that privacy during sessions, anonymity, and identity should be ensured in the use and storage of electronic materials |
| *Consent* | 1) Firm recommendations that documenting thorough consent processes consistent with relevant laws and regulations is important (58%, 11/19 guidelines) 2) Firm recommendations that these consent processes should address numerous issues unique to e-mental health services including privacy and confidentiality in the online domain, security steps taken, technological equipment and skills requirements, limits to communication, and reliability of the connection (63%, 12/19 guidelines)  3) Tentative recommendations that professionals should clarify contact information, and the nature of and expectations around therapeutic contact at the commencement of e-mental health interventions (47%, 9/19 guidelines) 4) Tentative recommendations that professionals should clarify expected timeframes for their client receiving a response from them, as well as processes around emergency contacts (47%, 9/19 guidelines) |
| *Professional boundaries* | 1) Tentative recommendations that mental health professionals should consider the increased potential for boundary issues to arise using e-mental health (21%, 4/19 guidelines)  2) Tentative recommendations that professionals should use the same level of professional language across all media as they would in person (21%, 4/19 guidelines) |
| *Crisis intervention and distress management* | 1) Firm recommendations that mental health professionals should establish in-person clinical supports in the client's geographic location prior to initiating e-mental health services, in case of emergency (53%, 10/19 guidelines) 2) Tentative recommendations that professionals should inform clients of alternative means of communication should the technology fail (42%, 8/19 guidelines) 3) Tentative recommendations that professionals should be familiar with mandatory reporting and involuntary hospitalisation laws (21%, 4/19 guidelines) |
| *Specific guidelines for certain populations* | High risk groups: Guidelines mentioned cognitive impairments and psychotic disorders as potential populations with a higher risk when using telehealth.  1) Some guidelines suggested it may be preferable to exclude these from e-mental health interventions, but one set of guidelines noted that there is no concrete evidence indicating which populations may benefit most or may be harmed by psychological therapy delivered via videoconferencing.  2) No recommendations were made regarding adaption for these groups.  Young people 1) Six guidelines discussed appropriateness of e-mental health services for young people. 42% (8/19 guidelines) highlighted importance of explicitly checking age in young people and their consent as they can appear highly adult due to high computer literacy.  2) All guidelines noted that the requirement for parental consent should remain for e-intervention.  3) Guidelines did not discuss tailored strategies for young people. |
